# Solvation Structure of Ag^+^ in 1‑Butyl-3-methylimidazolium Bis(trifluoromethylsulfonyl)imide Ionic Liquid: Evidence for Linear *N*‑Bound Coordination

**DOI:** 10.1021/acs.inorgchem.6c01404

**Published:** 2026-06-11

**Authors:** Matteo Busato, Martina Sanadar, Paola D’Angelo, Andrea Melchior

**Affiliations:** † 9311 Sapienza Università di Roma, Dipartimento di Chimica, p.le Aldo Moro 5, Roma 00185, Italy; ‡ Centre de Biophysique Moléculaire, CNRS UPR 4301, Université d’Orléans, rue Charles Sadron, Orléans 45071, France; § Università degli Studi di Udine, Dipartimento Politecnico di Ingegneria e Architettura, via delle Scienze 206, Udine 33100, Italy

## Abstract

The coordination environment of the Ag^+^ ion in the 1-butyl-3-methylimidazolium bis­(trifluoromethylsulfonyl)­imide ([C_4_mim]­[Tf_2_N]) ionic liquid (IL) was investigated through a combined experimental and theoretical approach including Raman and Fourier transform-infrared (FT-IR) spectroscopy, density functional theory (DFT) calculations, and X-ray absorption spectroscopy. Raman spectra in the 720–780 cm^–1^ region show the progressive conversion of “free” to coordinated [Tf_2_N]^−^ anions upon addition of the silver salt to [C_4_mim]­[Tf_2_N], and quantitative analysis indicates an average coordination number of approximately two anions per Ag^+^ ion. DFT calculations on the possible coordination isomers of the [Ag­(Tf_2_N)_2_]^−^ complex reveal that the most stable species consists of two monodentate [Tf_2_N]^−^ ligands bound to the Ag^+^ cation through the central nitrogen atom, in agreement with the characteristic shifts observed in the experimental FT-IR spectra. Extended X-ray absorption fine structure analysis supports the presence of a well-defined first coordination shell featuring two Ag–N interactions at 2.23(2) Å arranged in an almost linear N–Ag–N geometry. Overall, the combined results establish a predominantly linear, *N*-bound solvation motif for Ag^+^ in [C_4_mim]­[Tf_2_N]. This behavior contrasts with the coordination observed in other ILs and highlights the strong influence of the anion on metal ion solvation in these media.

## Graphical abstract

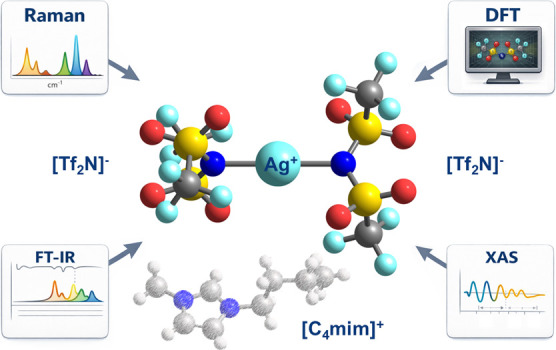

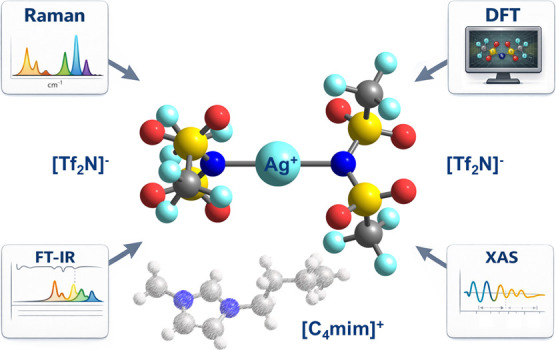

## Introduction

Over the past decades, ionic liquids (ILs) have attracted considerable interest as alternative solvents owing to their negligible vapor pressure, nonflammability, high thermal and electrochemical stability, and remarkable solvating ability.^1^ These features have enabled their application in areas such as selective extraction processes,^2–6^ energy storage devices,^7–10^ electrodepositions,^11–13^ and catalysis.^14,15^ In many of these contexts, metal salts are present as dissolved species in the IL phase, making the understanding of metal ion speciation and coordination structure a key issue for controlling reactivity, thermodynamic stability, and transport properties in these media.^16–23^

Among transition metals, silver holds particular technological relevance, and ILs have therefore been explored for the selective recovery of Ag^+^ from complex aqueous matrices^24–28^ as well as for electrodeposition processes of this metal.^12,29–31^ Solutions of Ag^+^ salts in ILs have been extensively investigated for selective olefin/alkane separation, a key step in hydrocarbon processing,^32^ exploiting the ability of Ag^+^ to selectively and reversibly bind olefins through π-complexation.^33–36^ However, despite this technological importance, the structural characterization of Ag^+^ in ILs remains significantly less explored than in conventional molecular solvents, whereas a clearer description of its solvation structure could support the understanding of the fundamental processes underlying these applications.

In conventional solvents, Ag^+^ exhibits a highly flexible coordination chemistry, which has been the subject of numerous structural^37–40^ and thermodynamic^41–47^ studies. In nonaqueous molecular solvents, Ag^+^ commonly adopts a tetrahedral coordination geometry,^37,48^ while in an aqueous solution, a distorted “2 + 2” structure, intermediate between tetrahedral and linear coordination, has been proposed based on X-ray absorption spectroscopy (XAS), large-angle X-ray scattering, and Car–Parrinello molecular dynamics (CPMD) simulations.^37,49^ Linear coordination, which is in principle possible due to orbital hybridization promoted by relativistic effects,^50^ has also been frequently observed in the presence of *N*-donor ligands in solution,^51,52^ highlighting the marked sensitivity of Ag^+^ coordination to the solvation environment.

Only a limited number of studies have addressed Ag^+^ solvation in ILs. CPMD simulations on 1-ethyl-3-methylimidazolium trifluoromethanesulfonate indicated a disordered and flexible solvation structure with coordination numbers (CNs) ranging from 2 to 5.^53^ In *N*-butyl-*N*-methylpyrrolidinium dicyanamide, attenuated total reflectance-ultraviolet spectroscopy, classical molecular dynamics (MD) simulations, and time-dependent density functional theory (DFT) calculations suggested an average of 4.5 anions around Ag^+^, consistent with a dynamic CN between 4 and 5.^54^ More recently, CPMD and XAS investigations on 1-butyl-3-methylimidazolium tetrafluoroborate ([C_4_mim]­[BF_4_]) demonstrated that Ag^+^ is coordinated by ∼4 [BF_4_]^−^ anions arranged in a roughly tetrahedral geometry.^23^ In this system, the coordinated anions were shown to be dynamically exchanging with the bulk IL, giving rise to the ${\left[Ag{\left({BF}_{4}\right)}_{4}\right]}^{3-}⇌{\left[Ag{\left({BF}_{4}\right)}_{3}\right]}^{2-}+{\left[{BF}_{4}\right]}^{-}$ equilibrium, while internal anion motions lead to a variable number (4–6) of coordinating fluorine atoms.

Particular attention deserve ILs based on the bis­(trifluoromethylsulfonyl)­imide anion ([Tf_2_N]^−^). These ILs can achieve high ionic conductivity and marked hydrophobic character, in particular, when paired with an imidazolium cation, making them attractive for electrochemical devices and liquid–liquid biphasic extractions from aqueous media.^7,55,56^ From a coordination chemistry perspective, [Tf_2_N]^−^ is known for its structural flexibility and its ability to coordinate metal ions in mono- or bidentate fashion through one or two oxygen atoms belonging to different sulfonyl groups, while coordination through the central nitrogen atom is less common. For divalent transition metal ions, coordination has been shown to occur predominantly via monodentate *O*-binding,^17–20,57^ although bidentate modes have also been proposed.^58–62^

For Ag^+^ in Tf_2_N-based ILs, however, the structural picture remains controversial. Raman and infrared (IR) spectroscopy studies on silver bis­(trifluoromethylsulfonyl)­imide (AgTf_2_N) dissolved in 1-ethyl-3-methylimidazolium bis­(trifluoromethylsulfonyl)­imide suggested an unusual coordination motif involving two [Tf_2_N]^−^ anions binding via oxygen and one via nitrogen atoms.^63^ The simulated vibrational spectra for this structure reproduced the experimental trends satisfactorily. However, alternative coordination motifs were not explored, preventing unambiguous discrimination among possible solvation structures. Coordination of sulfonyl imide anions through nitrogen finds precedent in solid-state structures containing the [Ag­(bis­[bis­(fluorosulfonyl)­imide)]^−^ and [Ag­(bis­[bis­(methanesulfonyl)­amide)]^−^ units.^64,65^ Here, the bis­(fluorosulfonyl)­imide and bis­(methanesulfonyl)­amide anions, which are structurally analogous to [Tf_2_N]^−^ but bearing –F and –CH_3_ instead of –CF_3_ groups, respectively, show coordination to Ag^+^ via nitrogen atoms in a linear geometry. In the Ag-based IL [Ag­(CH_3_CN)_4_]_2_[Ag­(Tf_2_N)_3_], the anionic unit contains Ag^+^ coordinated by three [Tf_2_N]^−^ ligands via nitrogen atoms.^30^ Furthermore, a 2-fold coordination is suggested by electrospray ionization-mass spectrometry of AgTf_2_N in 1-butyl-3-methylimidazolium bis­(trifluoromethylsulfonyl)­imide ([C_4_mim]­[Tf_2_N]) detecting the [Ag­(Tf_2_N)_2_]^−^ fragment, although with lower relative intensity compared to the corresponding divalent metal complexes, suggesting a more labile solvation structure for Ag^+^ in this IL.^60^ Such lability may also account for the different solvation thermodynamics shown by Ag^+^ in ILs compared to other metal ions. While the transfer of divalent metal ions like Co^2+^ and Zn^2+^ from water to [C_*n*_mim]­[Tf_2_N] (*n* = 2, 4) is a nonspontaneous process,^17,18^ Ag^+^ exhibits a marked different behavior. In particular, its transfer toward [C_4_mim]­[BF_4_] was found to be thermodynamically favorable, arising from an enthalpic–entropic balance where a disordered solvation structure and a reduced CN compared to divalent metals play a central role in solvation entropy.^23^ Furthermore, transfer-free energies for Ag^+^ seem to be only slightly unfavorable or even favorable toward Tf_2_N-based ILs, suggesting a completely different solvation environment compared to divalent metal cations.^23^

In this work, we investigate the coordination environment of the Ag^+^ ion in [C_4_mim]­[Tf_2_N] by combining Raman and Fourier transform-infrared (FT-IR) spectroscopy with XAS and DFT calculations. This integrated experimental–computational approach allowed us to resolve the local structure in the liquid phase and revealed an unusual coordination mode for the [Tf_2_N]^−^ anion, providing new insights into Ag^+^ speciation in Tf_2_N-based systems.

## Experimental Section

### FT-IR and Raman Spectroscopies

[C_4_mim]­[Tf_2_N] (99.5%, water content <100 ppm determined by Karl Fisher titration) and AgTf_2_N (99.5%) were purchased from IoLiTec GmbH (Germany) and used without further purification. All solutions were prepared inside a nitrogen-filled glovebox with water content <1 ppm. Samples with different metal salt concentrations were obtained by accurately weighing the required amounts of AgTf_2_N and IL. The Ag mole fraction (*x*) was varied in the range 0.0 ≤ *x* ≤ 0.40.

FT-IR spectra were recorded in attenuated total reflectance mode using a Thermo Fisher APEX instrument. Raman spectra were collected in the 100–1700 cm^–1^ range using an Xplora Plus Micro-Raman system (Horiba, Kyoto, Japan) equipped with a 785 nm laser source. The spectra were acquired at room temperature with a spectral resolution of 1 cm^–1^, using 10 accumulations of 20 s and a 50× LWD objective.

The Raman bands associated with free and coordinated [Tf_2_N]^−^ anions were deconvolved and analyzed following procedures previously reported in the literature.^61,66,67^ In particular, two bands in the 720–780 cm^–1^ region were used to distinguish between free and bound anions.^66,67^ The integrated area of the deconvolved band assigned to the free anion (*I*_F_) can be expressed as the product of the molar Raman scattering coefficient (*J*_F_) and the molar concentration of free [Tf_2_N]^−^ anion, *C*_F_ (*I*_F_ = *C*_F_·*J*_F_). Similarly, when the Ag mole fraction is used instead of molar concentration, *I*_F_ is given by^66^

1 $$I_{F}=J_{F}^{\prime }\cdot \left(1-n\cdot x\right)$$

where *n* is the number of coordinated anions and *J*_F_^′^ is the Raman “fractional” scattering coefficient.^66^ Then, eq 1 can be rewritten as eq 2, where *I*_0_ is the area of the Raman band in the absence of a metal ion:

2 $$\frac{I_{F}/I_{0}}{x}=J_{F}^{\prime }\cdot \left(\frac{1}{x}-n\right)$$

The number of coordinating anions *n* is obtained from linear regression using the slope and intercept parameters.

### DFT Calculations on Ag^+^ Complexes

DFT calculations were performed using the ωB97XD functional^68^ in combination with the def2-TZVP basis set.^69^ Geometry optimizations of the 1:2 [Ag­(Tf_2_N)_2_]^−^ complexes formed between Ag^+^ and [Tf_2_N]^−^ were first carried out in the gas phase and subsequently within a continuum solvent using the SMD model.^70^ The medium effects on the solutes were included by the SMD approach developed to reproduce the solvation free energies of a library of compounds.^71^ While this method does not take into account the heterogeneous nature of ILs, it provides a computationally convenient approach to include polarization effects on the solutes demonstrated to provide accurate results in the calculation of thermochemical data in solution.^72,73^ Vibrational analysis employing the harmonic oscillator approximation was performed for all the obtained minima and showed no imaginary frequencies. Additional geometry optimizations were carried out for the [Ag­(Tf_2_N)_2_]^−^ complexes including one [C_4_mim]^+^ counterion to include possible many-body effects in vibrational spectra.^63^ The [C_4_mim]­[Tf_2_N] ionic couple was also optimized at the same level of theory. The coordinates of minimum energy structures are reported in Supporting Information, while the complete vibrational frequencies and oscillator strengths are reported in Table S1. Free energies were calculated by adding to the electronic energy the zero-point vibrational energy (ZPVE) and thermal corrections. All calculations were performed using the Gaussian 16 program Rev. A.03.^74^

### XAS Measurements and Data Analysis

Ag K-edge XAS measurements were performed in transmission mode on a 0.1 mol L^–1^ AgTf_2_N solution in [C_4_mim]­[Tf_2_N] at the 11.1 XAFS beamline of Elettra–Sincrotrone Trieste (Italy).^75^ The liquid sample was loaded into a dedicated cell equipped with Kapton windows and maintained under a continuous nitrogen flux during data acquisition to prevent exposure to atmospheric moisture. Data were collected using a Si(311) double-crystal monochromator, with the storage ring operating at 2 GeV and a beam current between 200 and 300 mA. Energy calibration was performed using a silver metal foil as a reference. Three spectra were recorded and averaged to improve the signal-to-noise ratio.

The extended X-ray absorption fine structure (EXAFS) region of the absorption spectrum was analyzed using the GNXAS program.^76,77^ Scattering amplitudes and phase shifts were calculated within the muffin-tin (MT) approximation from clusters of fixed geometry. To this end, the DFT-optimized [Ag­(Tf_2_N)_2_]^−^ clusters were employed (see above). MT radii were chosen to ensure an ∼20% overlap between adjacent MT spheres and were set to 1.91, 1.00, 0.90, 0.80, 0.80, and 0.70 Å for Ag, S, O, N, C, and F atoms, respectively.^18,23,40^ Photoelectron inelastic losses in the final state were taken into account using advanced models for the exchange–correlation self-energy in the Hedin–Lundqvist scheme.^78^

Theoretical signals associated with *n*-body distribution functions were calculated within the multiple-scattering (MS) theory and summed to reconstruct the total theoretical contribution. In a first stage, the analysis was carried out by considering the N2 structure, identified as the most probable coordination in solution (*vide infra*). Accordingly, single-scattering (SS) theoretical signals involving the Ag–N, Ag–S, and Ag–O photoelectron paths were included, together with MS contributions associated with the Ag–N–S and N–Ag–N three-body distributions. The Ag–O path refers to the O atom closest to the photoabsorber. A schematic representation of the SS and MS paths included in the analysis is shown in Figure S1. Initial values for the N–S and S–O distances within the [Tf_2_N]^−^ anion were taken from the DFT-optimized structure (1.63 and 1.45 Å, respectively), as well as the Ag–N–O and N–S–O angles (118° and 108°, respectively), while a collinear configuration was considered for N–Ag–N. Each two-body distribution was modeled as a Γ-like function depending on four structural parameters: the path degeneracy *N*, the average distance *R*, the Debye–Waller factor σ^2^, and the asymmetry index β. These parameters were optimized during the fitting procedure, except for *N* values, which were fixed according to the N2 structural model. In addition to the N2 model, alternative coordination geometries identified from the combined IR/Raman and DFT analysis, namely, NO, O2, NO2, and O4, were also evaluated. For each model, theoretical SS and MS contributions were generated starting from the corresponding DFT-optimized cluster geometry and treated following the same fitting protocol described above. Least-squares minimizations were carried out over the 3.2–10.7 Å^–1^ *k*-range directly on the raw data, without preliminary background subtraction or Fourier filtering. In addition, nonstructural parameters were optimized, namely, the edge energy position *E*_0_ and the energy positions and amplitudes of the double-electron excitations KN_2&3_, KN_1_, and KM_4&5_. The amplitude reduction factor *S*_0_^2^ was constrained between 0.95 and 1.00.

## Results and Discussion

### Raman Spectroscopy

Raman spectra of AgTf_2_N solutions in [C_4_mim]­[Tf_2_N] at increasing metal concentrations were recorded to monitor the coordination of the [Tf_2_N]^−^ anion. As shown in Figure 1a, the most significant spectral changes upon metal salt addition occur in the 720–780 cm^–1^ region. This spectral range contains a diagnostic vibrational feature for [Tf_2_N]^−^ coordination, arising from the overlap between the –CF_3_ bending modes and the S–N–S stretching, commonly referred to as the [Tf_2_N]^−^ “breathing mode”.^61,63,66^ In neat [C_4_mim]­[Tf_2_N], this band is centered at 740 cm^–1^, and its intensity progressively decreases upon increasing the Ag mole fraction, while a new band appears at 752 cm^–1^ (Figure 1b). The two bands evolve with respect to a well-defined isosbestic point, indicating an equilibrium between two dominant populations in solution, namely, free and coordinated [Tf_2_N]^−^.

**Figure 1:**
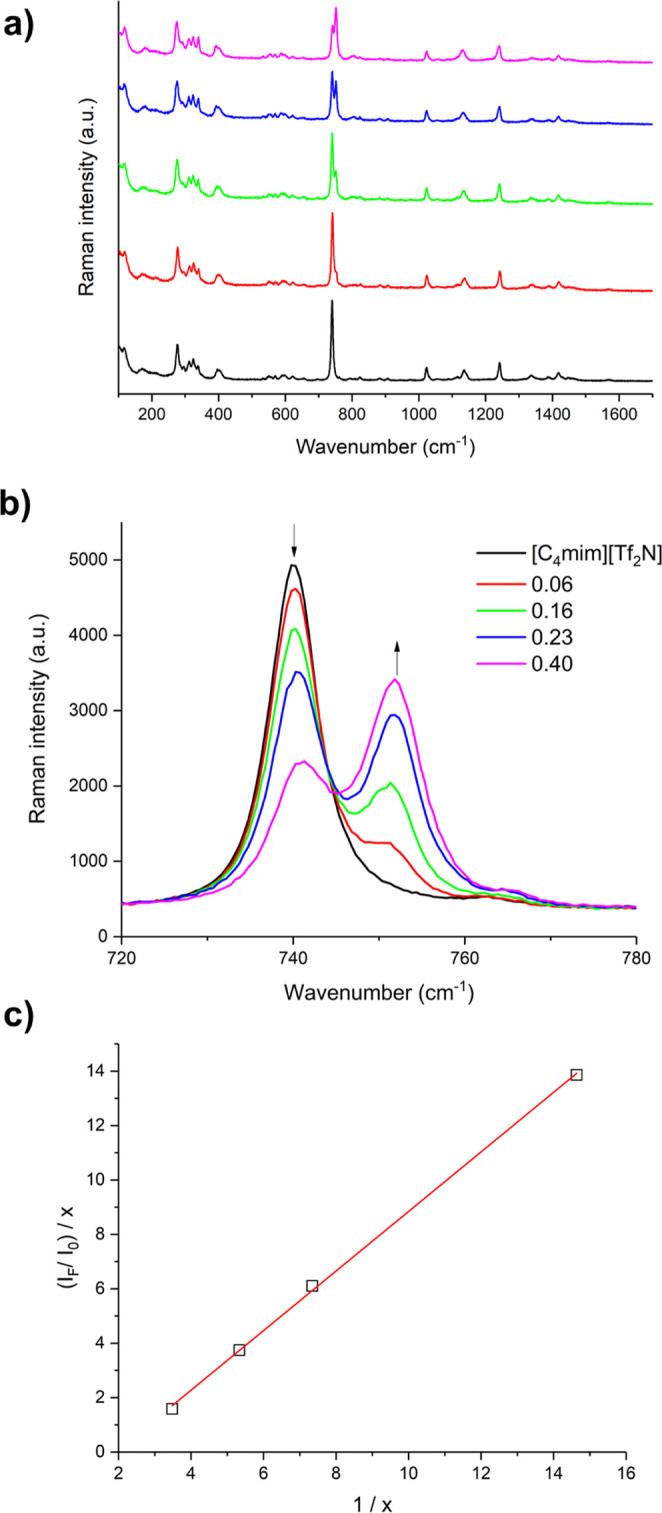
Raman spectra of AgTf_2_N solutions in [C_4_mim]­[Tf_2_N] at an increasing (0.0–0.4) metal salt mole fraction (*x*) in the (a) 100–1700 cm^–1^ and (b) 720–780 cm^–1^ wavenumber ranges. (c) (*I*_F_/*I*_0_)/*x* vs 1/*x* plot of deconvolved Raman bands in the 720–780 cm^–1^ region. *R* ^2^ = 0.999.

The deconvolution of the overlapping contributions (Figure S2) allows separation of the bands associated with free and bound anions. This yields the plot in Figure 1c and, using eq 2, a value of *n* = 1.92 ± 0.15 is determined. For comparison, the same data set was analyzed using the approach proposed by Lasségues et al.,^79^ which assumes identical Raman scattering coefficients for the free and uncoordinated anion bands (Figure S3). This procedure provides a value of *n* = 2.22 ± 0.05. Despite the different underlying assumptions, both methods consistently indicate that approximately two [Tf_2_N]^−^ anions are coordinated to Ag^+^ in solution.

### FT-IR and DFT Results

To assess the coordination mode of the [Tf_2_N]^−^ anions toward Ag^+^, DFT calculations were performed by exploring all plausible geometries of the [Ag­(Tf_2_N)_2_]^−^ complex. Five distinct coordination isomers were identified depending on the binding fashion of the anion. In the N2 structure, both [Tf_2_N]^−^ anions bind Ag^+^ through the central nitrogen atoms. In the NO isomer, a mixed coordination is obtained, with one anion binding through nitrogen and the other through oxygen in a monodentate fashion. The O2 structure corresponds to bis *O*-monodentate coordination, whereas in the NO2 isomer, one anion coordinates via nitrogen, while the second adopts an *O*-bidentate mode. Finally, in the O4 structure, both anions bind in a bidentate fashion through oxygen atoms. In addition, it should be considered that the [Tf_2_N]^−^ anion is known to exist in two conformations, namely, a transoid (trans) and cisoid (cis), depending on the relative orientation of the—CF_3_ groups with respect to the S–N–S plane.^67,80^ Both cis and trans conformers of the [Tf_2_N]^−^ anion were optimized at the DFT level for the isolated anion and for the [C_4_mim]­[Tf_2_N] ionic couple (Figure S4), evidencing a small energy difference (∼0.3 kcal mol^–1^ for the ionic couples), which is compatible with a conformational equilibrium shifted toward the trans form in the neat IL.^81^ Since both rotamers can coordinate to a metal center, preliminary optimizations were performed for the N2 structure, considering both the cis,trans and cis,cis arrangements of the two coordinated anions (Figure S5), and their energy was compared to the trans,trans form (N2 in Figure 2). The trans,trans conformation was found to be the more stable one than the others (1.4 and 4.9 kcal mol^–1^, respectively). Also, the FT-IR spectra in the 450–700 cm^–1^ region of [C_4_mim]­[Tf_2_N] solutions containing different amounts of Ag salt (Figure S6) display a small, but appreciable, change in intensity with a decrease of the peaks at 599 and 652 cm^–1^ and an increase at 612 cm^–1^, which have been previously^79^ assigned to the cis (599 and 652 cm^–1^) and trans (612 cm^–1^) conformers of the [Tf_2_N]^−^ anion in the pure ILs. Thus, the relative intensity change suggests a shift of the conformational equilibrium toward the trans form induced by the coordination to the metal ion. Based on these results, and to limit the number of species to be considered for the DFT calculations, only the trans,trans arrangement for the coordinated anions was adopted for the coordination isomers considered in this work.

**Figure 2:**
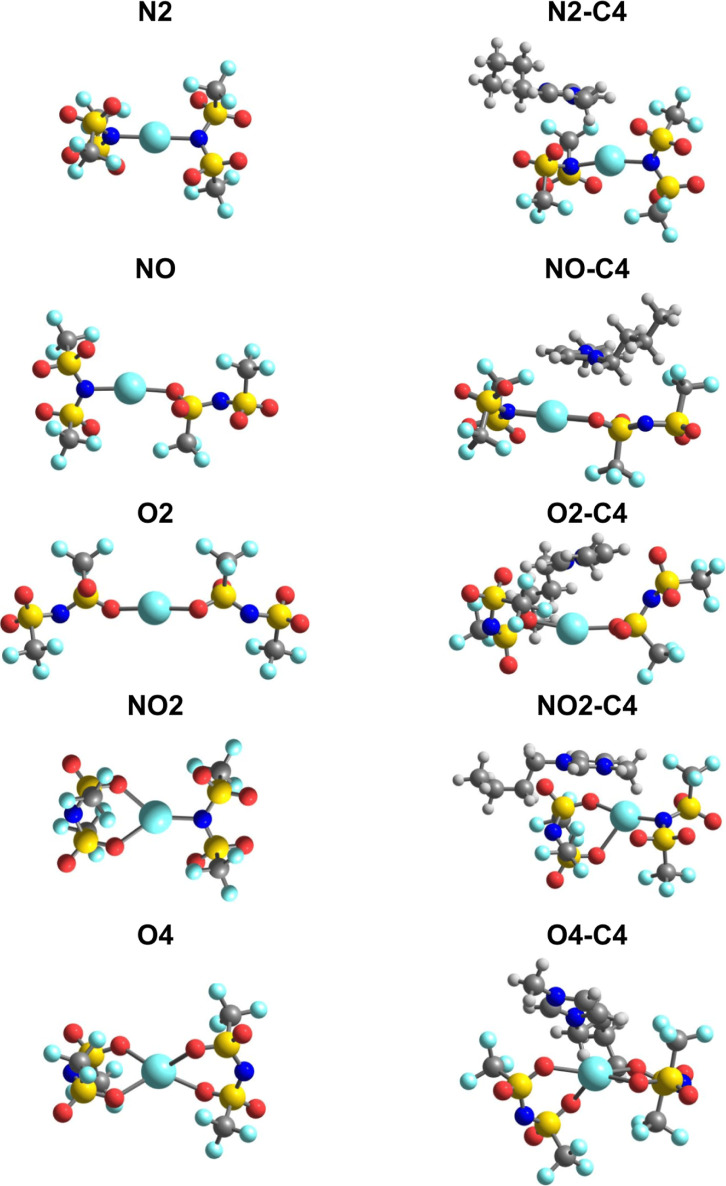
Minimum energy structures of the possible coordination isomers of [Ag­(Tf_2_N)_2_]^−^ (left) and of the [C_4_mim]­[Ag­(Tf_2_N)_2_] ionic couples (right) at the ωB97XD/def2-TZVP level in the SMD solvent.

The minimum energy structures of the possible isomers for both the [Ag­(Tf_2_N)_2_]^−^ complexes and the [C_4_mim]­[Ag­(Tf_2_N)_2_] ionic couples are shown in Figure 2, while the corresponding Ag–N and Ag–O bond distances are collected in Table S2. The relative stability of the isomers was evaluated in terms of free energy differences, defined as Δ*G*_rel_ = *G*_isomer_ – *G*_N2_ (or *G*_N2–C4_ for the ionic couples), where the N2 (or N2–C4) structure is taken as the reference. The resulting values are reported in Table 1 for both gas phase and continuum solvent conditions. For the [Ag­(Tf_2_N)_2_]^−^ complexes in solvent, the stability order is N2 > NO2 > O4 ∼ NO > O2. A similar trend is obtained for the [C_4_mim]­[Ag­(Tf_2_N)_2_] ionic couples, with the order N2–C4 > NO2–C4 ∼ NO–C4 > O4–C4 > O2–C4. In both series, the N2 (and N2–C4) isomer is predicted to be the most stable in both gas phase and in continuum solvent, with an energy difference of 3.4 kcal mol^–1^ (N2) and 3.8 kcal mol^–1^ (N2–C4) relative to the next most stable species in solvent (NO2/NO2–C4, Table 1). Such non-negligible energy gaps suggest the possible predominance of the N2 coordination motif in solution.

**Table 1:** Relative Free Energies (Δ*G*_rel_, kcal mol^–1^) of the [Ag­(Tf_2_N)_2_]^−^ Isomers and the [C_4_mim]­[Ag­(Tf_2_N)_2_] Ionic Couples in the Gas Phase and in SMD Solvent

isomer	gas	solv	isomer	gas	solv
N2	0.0	0.0	N2–C4	0.0	0.0
NO	6.8	6.1	NO–C4	2.2	5.3
O2	13.7	11.2	O2–C4	7.1	9.8
NO2	1.2	3.4	NO2–C4	3.2	3.8
O4	3.5	5.4	O4–C4	0.7	5.7

The experimental FT-IR spectra in the 950–1450 cm^–1^ region (Figure 3) show clear and systematic changes upon increasing the Ag molar fraction. In particular, when AgTf_2_N is dissolved, the intense band at 1051 cm^–1^ present in the neat IL (black spectrum in Figure 3) decreases in intensity and, simultaneously, a new feature appears at 1004 cm^–1^. At the same time, the absorbance at 1132 cm^–1^ slightly increases.

**Figure 3:**
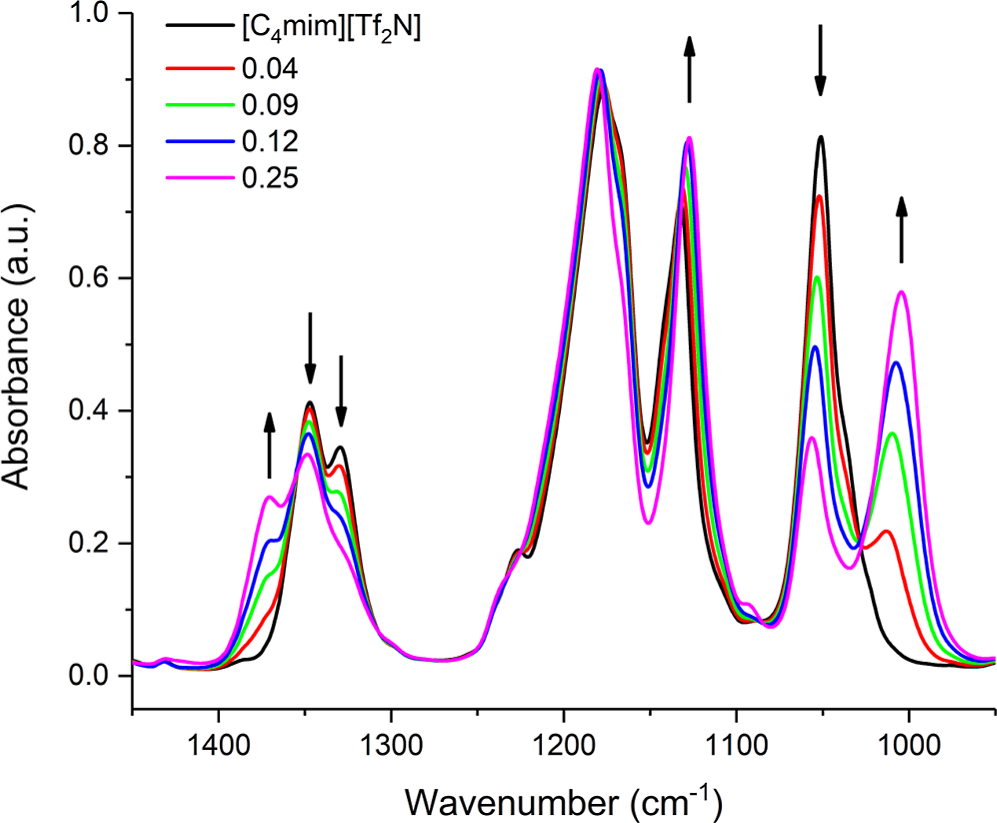
Experimental FT-IR spectra of AgTf_2_N solutions in [C_4_mim]­[Tf_2_N] at increasing *x* (0.0–0.25). Black arrows indicate the direction of peak changes upon addition of AgTf_2_N.

The vibrational analysis of the minimum-energy structure of the [C_4_mim]­[Tf_2_N] ionic couple (black spectrum in Figure 4) predicts two intense bands at 1078 and 1200 cm^–1^ (the latter composed by two components at 1200 and 1173 cm^–1^), associated with asymmetric S–N–S stretching modes (Table S1). The calculated frequencies are in agreement with the experimental bands at 1051 and 1132 cm^–1^ (Figure 3), which were also previously assigned to S–N–S vibrations.^63^ The calculated IR spectra (Figure 4) for the [C_4_mim]­[Ag­(Tf_2_N)_2_] complexes in Figure 2 differ markedly from that of the [C_4_mim]­[Tf_2_N] ionic couple, consistent with the experimental changes observed upon addition of the metal salt to the neat IL (Figure 3). Notably, the isomers in which the [Tf_2_N]^−^ anion coordinates via the nitrogen atom (N2–C4, NO–C4, and NO2–C4) display a new S–N–S stretching band at lower wavenumbers with respect to that calculated for the [C_4_mim]­[Tf_2_N] ionic couple (Figure 4 and Table S1). The calculated maxima centered at 1015 cm^–1^ for N2–C4, NO–C4, and NO2–C4 are in good agreement with the new experimental band appearing at 1004 cm^–1^ upon metal salt dissolution. Importantly, this feature is absent for the isomers where coordination occurs exclusively through oxygen atoms (O2–C4 and O4–C4, dashed spectra in Figure 4). This downshift of the S–N–S stretching frequency therefore emerges as a spectral fingerprint of *N*-coordination of the [Tf_2_N]^−^ anion. Moreover, the calculated spectra of the *O*-coordinated species predict intense bands at 1082 cm^–1^ (O4–C4) and 1078 cm^–1^ (O2–C4), which are not detected experimentally. The N2–C4 structure is the only isomer that does not present spectral features in this region. Altogether with the relative stabilities in Table 1, these observations suggest that O-coordinated species are unlikely to be present in significant amounts.

**Figure 4:**
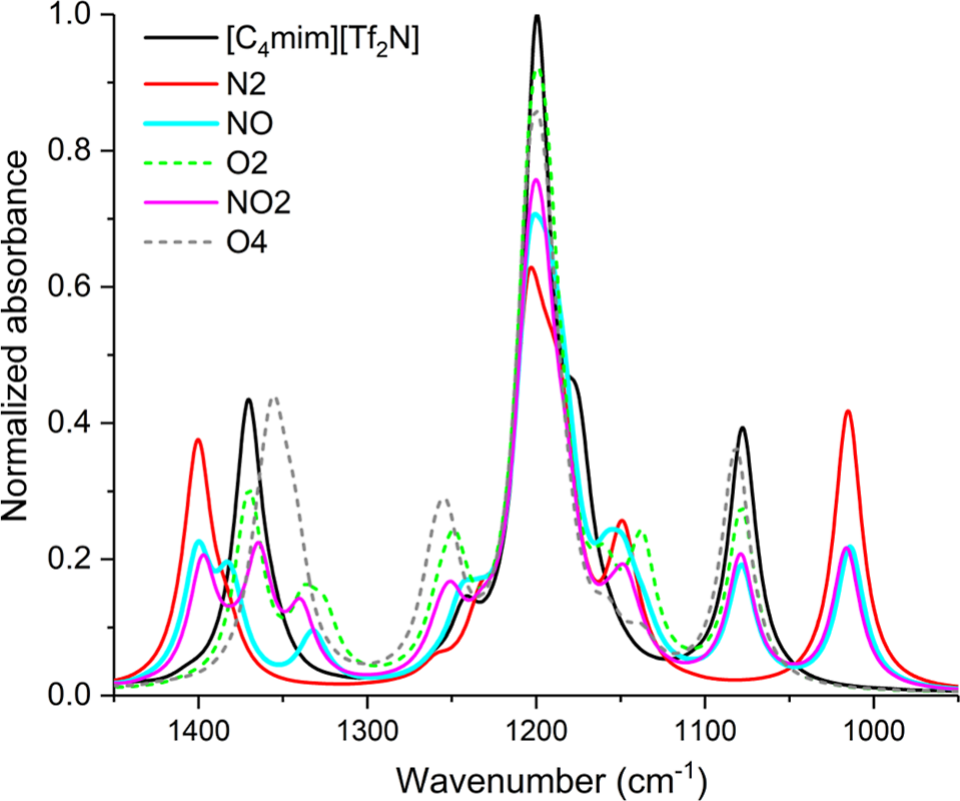
Calculated FT-IR spectra in the 950–1450 cm^–1^ range for [C_4_mim]­[Tf_2_N] and for the [C_4_mim]­[Ag­(Tf_2_N)_2_] complexes, computed at the ωB97XD/def2-TZVP level in the SMD solvent. Vibrational wavenumbers were scaled by a factor of 0.9914 ^82^ and spectral bands were generated using Gaussian functions with a 10 cm^–1^ half-width at half-maximum.

Additional evidence arises from the 1300–1400 cm^–1^ region. In the experimental spectrum of the neat IL, a band with two maxima at 1347 and 1330 cm^–1^ decreases in intensity upon addition of the metal salt, while a new band appears at 1371 cm^–1^ (Figure 3). The calculated spectrum of [C_4_mim]­[Tf_2_N] presents a single band centered at 1370 cm^–1^, resulting from the combination of two vibrational modes at 1370 and 1355 cm^–1^, assigned to S=O stretching motions (Figure 4 and Table S2). In the calculated spectra of the N2–C4 isomer, this band shifts to higher wavenumbers, with a calculated maximum at 1400 cm^–1^. Similarly, NO–C4 and NO2–C4 display weaker and shifted bands at 1400 and 1398 cm^–1^, respectively. In contrast, the spectrum of O2–C4 exhibits a new band centered at 1335 cm^–1^, corresponding to the stretching of uncoordinated S=O groups. In the case of O4–C4, this feature is located at 1356 cm^–1^ with a shoulder at 1343 cm^–1^. The overall results show that only *N*-coordinated species produce a new band at higher wavenumbers relative to [C_4_mim]­[Tf_2_N], in agreement with the experimental spectra showing a new band at 1371 cm^–1^ upon dissolution of the silver salt (Figure 3). Furthermore, no new bands appear below 1330 cm^–1^ in the experimental spectra, whereas such features are predicted for *O*-coordinated structures (dashed spectra in Figure 4).

Overall, the comparison between experimental and calculated FT-IR spectra (Figures 3 and 4), together with the computed relative free energies (Table 1), consistently supports that the Ag^+^ ion predominantly adopts a linear coordination with two [Tf_2_N]^−^ anions bound through the central nitrogen atom, with *O*-bound species being disfavored.

### XAS Results

The EXAFS analysis of the 0.1 mol L^–1^ AgTf_2_N solution in [C_4_mim]­[Tf_2_N] was performed to obtain a structural description of the local coordination environment of the Ag^+^ ion. The N2 structural model was tested to confirm the presence and predominance of this species in solution. The best-fit results are shown in Figure 5. In the upper panel, the Ag–N, Ag–S, and Ag–O SS theoretical signals, as well as the Ag–N–S and N–Ag–N MS ones, are reported together with their sum into the total theoretical contribution, which is compared to the experimental data and the resulting residuals. A good agreement between the calculated and the experimental spectra is observed, as further confirmed by the corresponding Fourier transforms (FTs) reported in the lower panel of Figure 5, calculated over the 3.2–10.0 Å^–1^ *k*-range. The optimized *E*_0_ value was found to be 2.7 eV above the first inflection point of the experimental spectrum, while *S*_0_^2^ was 0.97.

**Figure 5:**
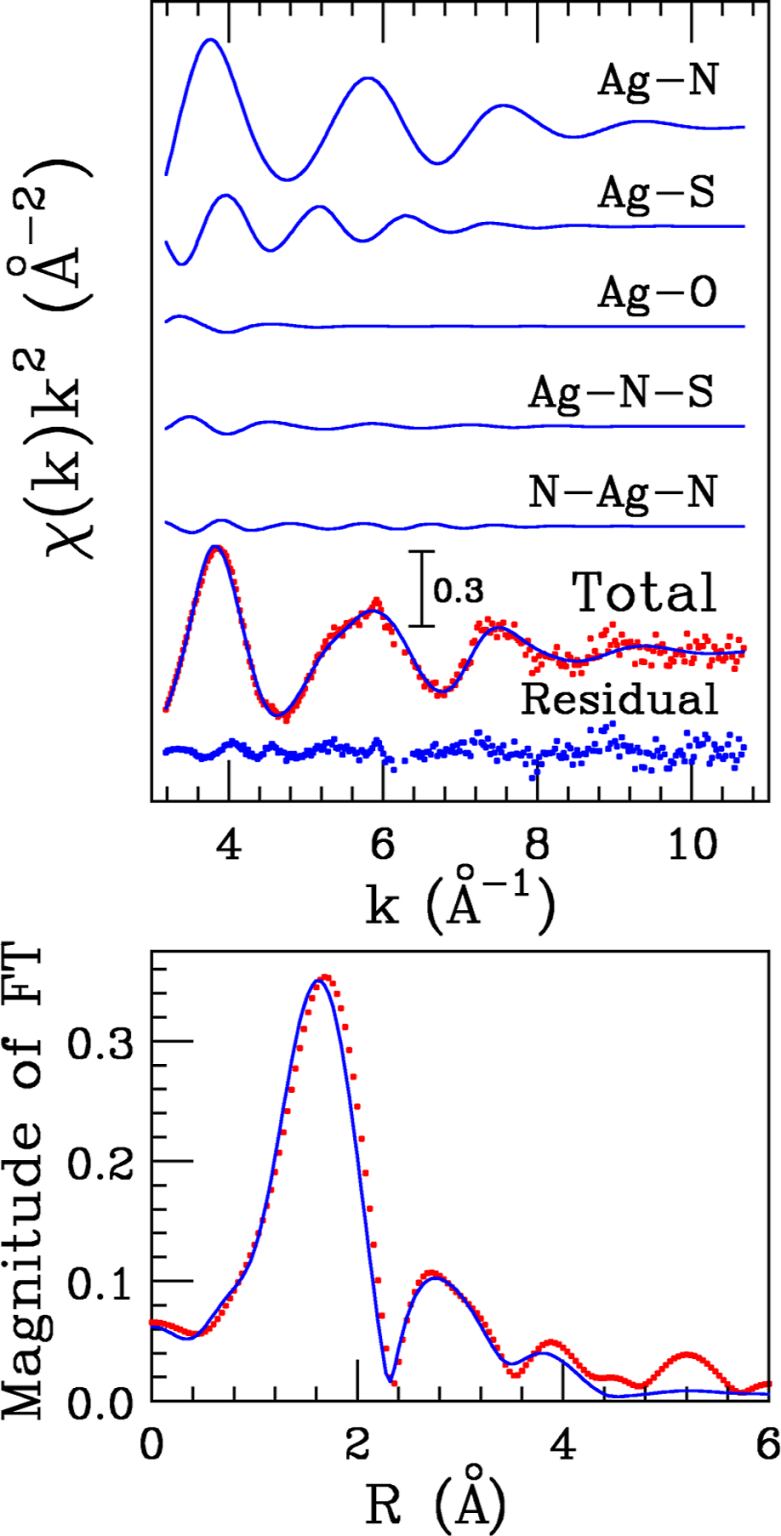
Upper panel: Ag K-edge EXAFS analysis of the 0.1 mol L^–1^ AgTf_2_N solution in [C_4_mim]­[Tf_2_N]. From top to bottom: Ag–N, Ag–S, and Ag–O SS theoretical signals, Ag–N–S and N–Ag–N MS theoretical signals, and total theoretical spectrum (blue lines) compared with the experimental one (red dots) and resulting residuals (blue dots). Lower panel: nonphase-shift-corrected FTs of the total theoretical signal (blue line) and of the experimental data (red dots).

The structural parameters determined for the two-body distributions are listed in Table 2, while the refined values for the Ag–N–S and N–Ag–N bond angles resulted in 118(6)° and 179(5)°, respectively. The first-shell contribution is dominated by the Ag–N signal, with an average distance of 2.23(2) Å, corresponding to the main peak in the FT. This value is in good agreement with the Ag–N distance obtained from the DFT-optimized structure (2.209 Å, Table S2), within the experimental uncertainty. The Ag–S signal is also marked in amplitude, reflecting the strong backscattering contribution of sulfur atoms located at an average distance of 3.31(3) Å, and dominates the second FT peak centered at about 3 Å. In contrast, the MS contributions and the Ag–O signal from the more distant oxygen atoms at 3.35(4) Å are weaker in intensity. The relatively small σ^2^ value for the Ag–N distribution indicates a well-defined first coordination shell, whereas the larger σ^2^ values associated with Ag–S and Ag–O reflect increased structural disorder at longer distances, consistent with the intrinsic flexibility of a structurally complex anion such as [Tf_2_N]^−^.^17,18,49,57^ However, this interpretation must take into account the well-known correlation between path degeneracies and Debye–Waller factors in the EXAFS region and the uncertainty that arises in the determination of these parameters. Overall, the refined bond distances and angles are consistent with the N2 geometry, and the EXAFS analysis is therefore compatible with this coordination motif of the Ag^+^ ion in the IL solution.

**Table 2:** Best-Fit Structural Parameters for the Ag–N, Ag–S, and Ag–O Two-Body Distributions Obtained from the Ag K-Edge EXAFS Analysis of the 0.1 mol L^–1^ AgTf_2_N Solution in [C_4_mim]­[Tf_2_N]. *N* Is the Path Degeneracy, *R* the Average Distance, σ^2^ the Debye–Waller Factor, and β the Asymmetry Index

	*N*	*R* (Å)	σ^2^ (Å^–2^)	β
Ag–N	2.0	2.23(2)	0.009(2)	0.0(1)
Ag–S	4.0	3.31(3)	0.033(4)	0.0(2)
Ag–O	4.0	3.35(4)	0.082(5)	0.0(3)

To evaluate alternative possible geometries, additional EXAFS fits were also performed for the NO, O2, NO2, and O4 models. The results are shown in Figure S7, where the total theoretical EXAFS signals for each geometry are compared with the experimental data (Figure S7a), together with the corresponding FTs (Figure S7b). Alternative models generally show progressively poorer agreement with both the experimental EXAFS signal and FT, while the comparison with the fit obtained for the N2 model (Figure 5) indicates that the N2 structure provides the best agreement with the experimental data. The NO model yields a partially satisfactory description, consistent with its retention of one Ag–N interaction, whereas *O*-dominated and higher-coordination models progressively worsen the agreement. In particular, the models with first-shell CNs greater than two, such as NO2 and O4, tend to overestimate the EXAFS amplitude and the intensity of the first FT peak. The overall results indicate that, although minor contributions from alternative structural motifs cannot be completely ruled out, particularly mixed O/N coordinations like NO or NO2, the EXAFS analysis supports N2 as the dominant species under the investigated conditions. Taken together, these findings point to a specific coordination behavior of the Ag^+^ ion in solution that differs markedly from that observed for other divalent transition metals^17–20,57–62^ and also for monovalent ions such as Li^+^ or Na^+^,^67,83^ which present the anion coordinated exclusively via oxygen in monodentate or chelating modes. This behavior is in agreement with the HSAB theory, which predicts a preferential interaction of the soft acid Ag^+^ with the soft base imide nitrogen atom^51^ and has previously been identified in the solid state also for other soft metal ions (Cu^+^ and Au^+^).^84,85^

## Conclusions

In this work, the solvation structure of the Ag^+^ ion in the [C_4_mim]­[Tf_2_N] IL has been elucidated through the combination of Raman and FT-IR spectroscopy, DFT calculations, and XAS. Raman measurements in the 720–780 cm^–1^ region reveal the progressive conversion of free to coordinated [Tf_2_N]^−^ anions upon addition of AgTf_2_N. Quantitative analysis of the Raman bands indicates that, over the investigated concentration range, each Ag^+^ ion is coordinated by two [Tf_2_N]^−^ anions.

DFT calculations allowed the discrimination among possible [Ag­(Tf_2_N)_2_]^−^ coordination isomers. The computed relative free energies and vibrational signatures, in direct comparison with the evolution of the experimental FT-IR spectra, consistently identify a predominant complex in which Ag^+^ is linearly coordinated by two monodentate [Tf_2_N]^−^ ligands through the central nitrogen atom. In contrast, *O*-bound coordination modes are both energetically disfavored and spectroscopically inconsistent with the experimental observations, suggesting that their contribution, if present, is minor.

The EXAFS analysis provides a structural validation of this model, revealing a well-defined first coordination shell characterized by two Ag–N interactions at 2.23(2) Å and an almost collinear N–Ag–N arrangement. Although the presence of minor species in equilibrium cannot be completely ruled out, the combined experimental and theoretical evidence clearly identifies the linear *N*-bound motif as the dominant solvation structure of Ag^+^ in [C_4_mim]­[Tf_2_N].

To the best of our knowledge, these results provide the first unambiguous structural evidence of linearly coordinated Ag^+^ in an ionic liquid. The marked contrast with the coordination structures previously observed in molecular solvents and in other ILs highlights the strong sensitivity of Ag^+^ speciation to the solvation environment and underscores the critical role of the anion in dictating the metal ion solvation structure.

## Supplementary Material



## Notes

Schematic representation of EXAFS single and multiple scattering paths; Raman band deconvolution and analysis of the 720–780 cm^–1^ region; optimized geometries of the conformers of the [Tf_2_N]^−^ anion and [C_4_mim]­[Tf_2_N] ionic couple and the [Ag­(Tf_2_N)_2_]^−^ complexes; 450–700 cm^–1^ region of the experimental FT-IR spectra; EXAFS analysis using alternative coordination models; calculated vibrational frequencies and intensities for ionic species and complexes; calculated Ag–N and Ag–O bond lengths for optimized structures; and Cartesian coordinates of the obtained minimum energy structures (PDF)
