# Supplementary material for: Solvation Structure of Ag+ in 1‑Butyl-3-methylimidazolium Bis(trifluoromethylsulfonyl)imide Ionic Liquid: Evidence for Linear N‑Bound Coordination
Source: Inorg Chem. 2026 Jun 11;65(25):14023–32. doi: 10.1021/acs.inorgchem.6c01404 (PMC13321300; doi:10.1021/acs.inorgchem.6c01404)
Supplement: Supplementary file 1 [file ic6c01404_si_001.pdf]

Solvation structure of Ag^+ in 1-butyl-3-methylimidazolium bis(trifluoromethylsulfonyl)imide ionic liquid: evidence for linear N-bound coordination

Matteo Busato,† Martina Sanadar, ‡ Paola D'Angelo,† Andrea Melchior*⁋

†*Dipartimento di Chimica, Sapienza Università di Roma, p.le Aldo Moro 5, 00185, Roma, Italy*

‡*Centre de Biophysique Moleculaire, CNRS UPR 4301, Université d'Orléans, rue Charles Sadron, 45071, Orléans, France*

⁋*Dipartimento Politecnico di Ingegneria e Architettura, Università di Udine, via delle Scienze 206, 33100, Udine, Italy*

Supporting Information

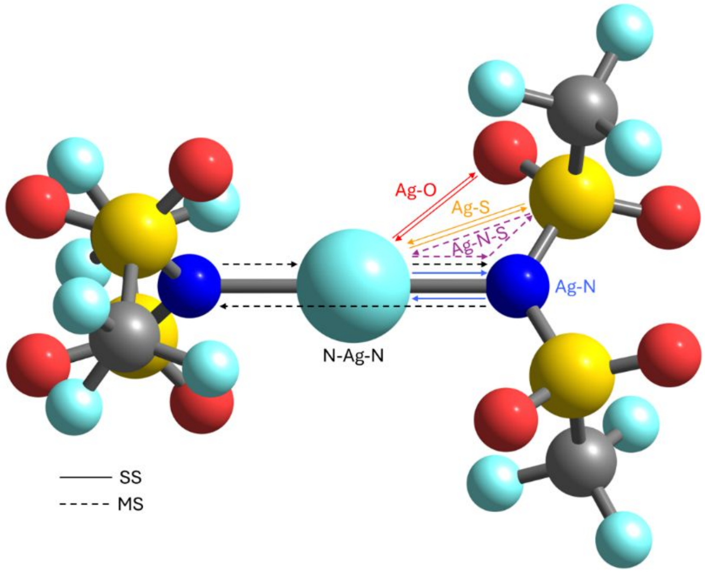

Figure S1. Schematic representation of the single-scattering (SS, solid lines) and multiple-scattering (MS, dashed lines) paths included in the Ag K-edge EXAFS analysis of the 0.1 mol L^{-1} $AgTf_2N$ solution in $[C_4mim][Tf_2N]$. Arrows are shown for clarity only and do not account for path degeneracy.

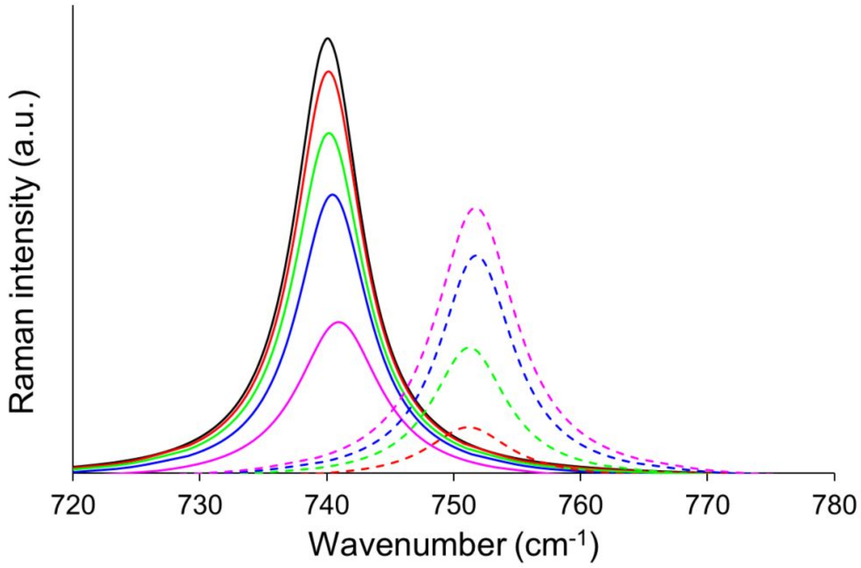

Figure S2. Deconvolution of the Raman bands in the 720 - 780 cm^{-1} region for $AgTf_2N$ solutions in $[C_4mim][Tf_2N]$ at increasing metal salt mole fraction x = 0.0 (black), 0.06 (red), 0.16 (green), 0.23 (blue), and 0.40 (pink). Lorentzian functions have been used for peak fitting.

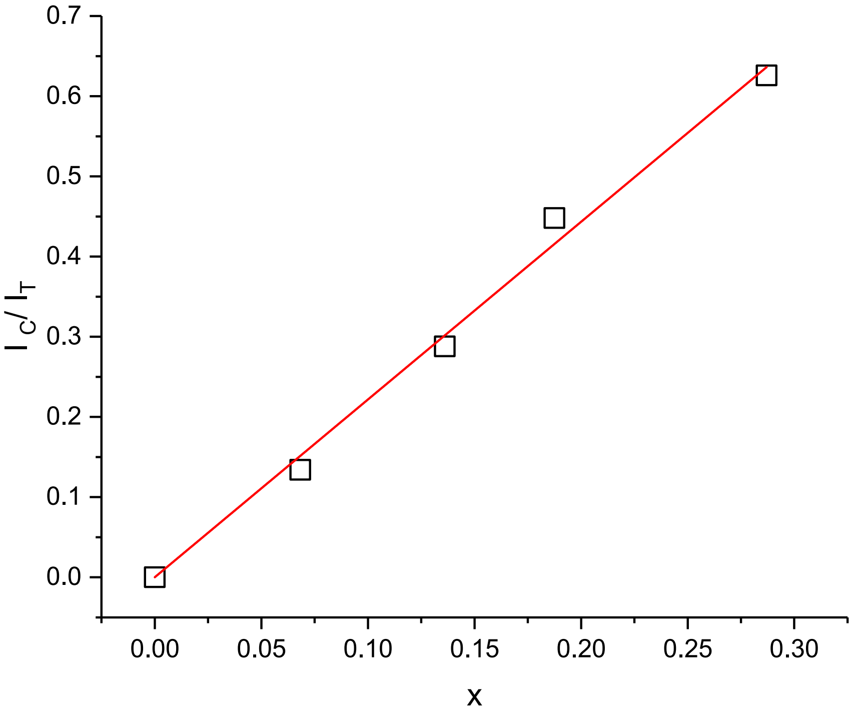

Figure S3. I_C/I_T *vs*. *x* plot of deconvoluted Raman bands in the 720 – 780 cm^{-1} region (Figure S2), used to determine the number of coordinated $[Tf_2N]^-$ anions, as described by Lassegues *et al*., *J. Phys. Chem. A* 2009, 113, 305–314. I_C: area of the deconvoluted band centered at 752 cm^{-1} (coordinated anion). I_T: total area of the bands assigned to coordinated and free anions ($I_T = I_C + I_F$). R^2 = 0.997.

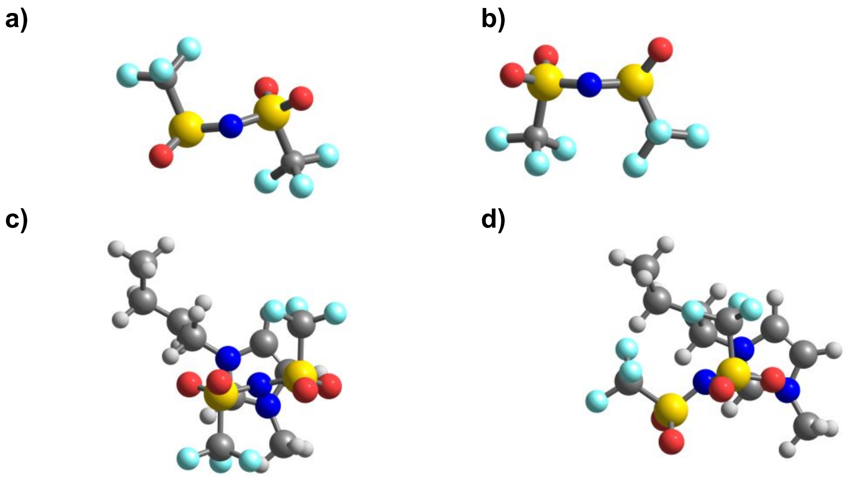

Figure S4. Minimum energy structures of the *trans* and *cis* conformers of the $[Tf_2N]^-$ anion (a,b , ΔG_{rel} (cis-trans) = -0.5 kcal mol^{-1}) and of the corresponding $[C_4mim][Tf_2N]$ ionic couples (c,d ΔG_{rel} (cis-trans)= 0.3 kcal mol^{-1})), optimized at the ωB97XD/def2-TZVP level.

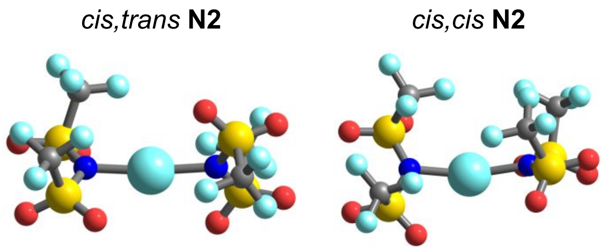

Figure S5. Minimum energy structures of the $[Ag(Tf_2N)_2]^-$ complex in the N2 coordination geometry, with the $[Tf_2N]^-$ ligands adopting *cis,trans* and *cis,cis* conformations, optimized at the ωB97XD/def2-TZVP level. The relative free energies (ΔG_{rel}) referenced to the *trans,trans* **N2** conformer are 1.4(0.9) and 4.9(4.1) kcal mol^{-1} for the *cis,trans* and *cis,cis* conformers, respectively. Values in parentheses were obtained using the SMD solvent model.

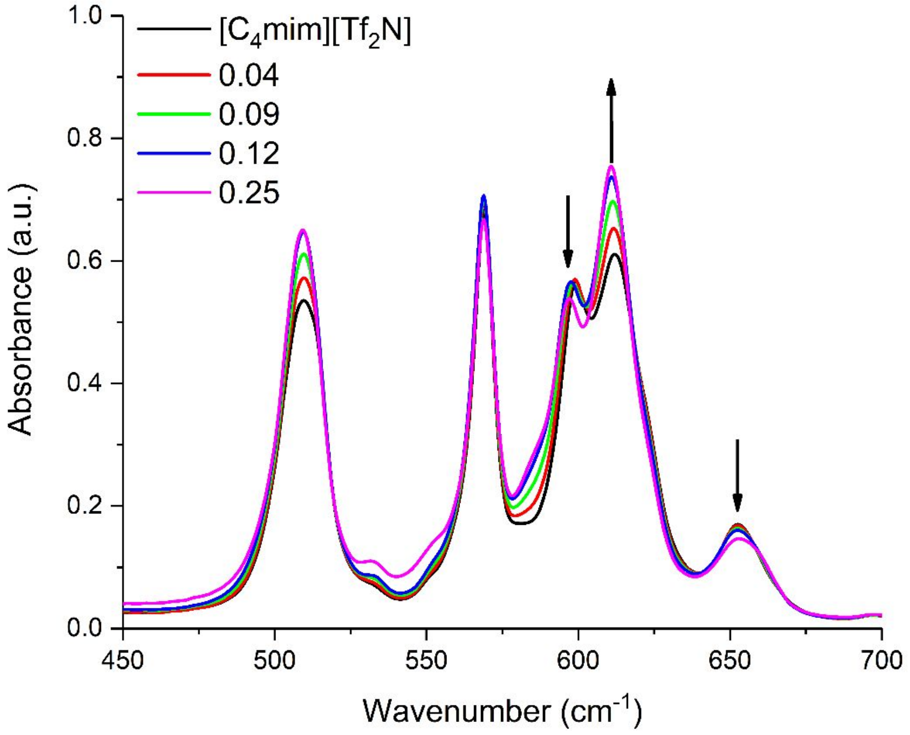

Figure S6. 450-700 cm^{-1} region of the experimental FT-IR spectra of $AgTf_2N$ solutions in $[C_4mim][Tf_2N]$ at increasing metal salt mole fractions. Black arrows indicate the direction of peak changes upon additions of the salt.

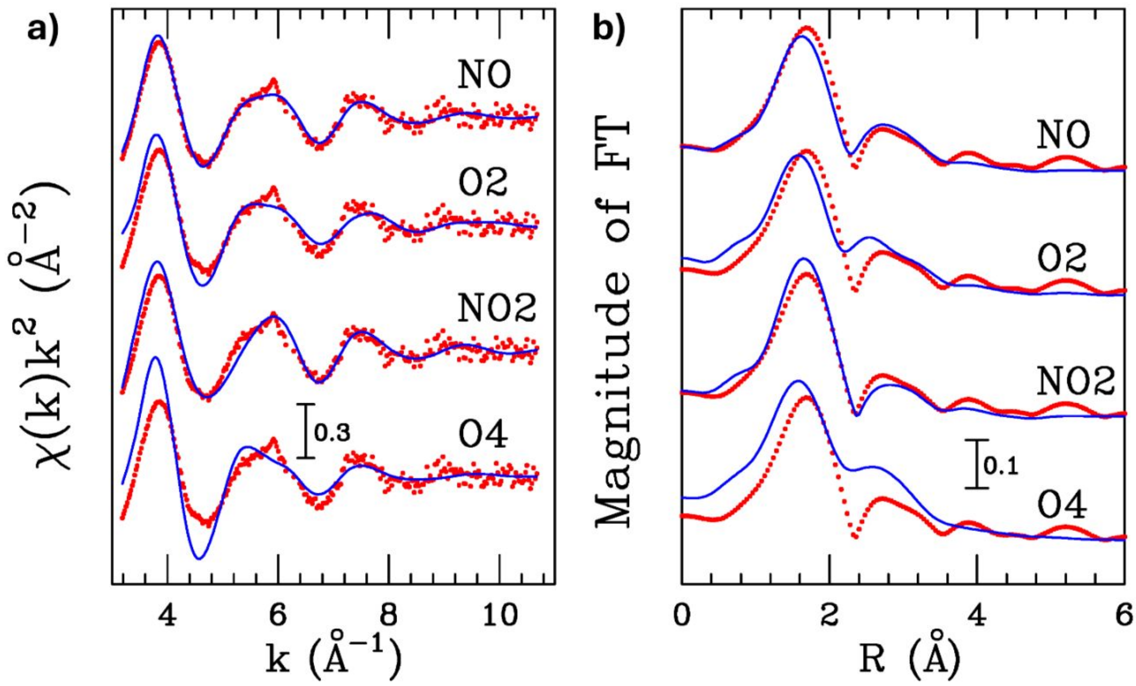

Figure S7. Ag K-edge EXAFS analysis of the 0.1 mol L^{-1} $AgTf_2N$ solution in $[C_4mim][Tf_2N]$ using alternative coordination models. a) Experimental EXAFS spectrum (red dots) compared with the total theoretical signals (blue lines) calculated for the NO, O2, NO2, and O4 coordination motifs. b) Corresponding non-phase-shift-corrected Fourier transforms of the experimental data (red dots) and of the theoretical signals (blue lines).

Table S1. Vibrational frequencies (unscaled, **freq**, cm^{-1}) and calculated intensities (**I**, km/mol) for the $[Tf_2N]^-$ and $[Ag(Tf_2N)_2]^-$ anions and for the $[C_4mim][Tf_2N]$ and $[C_4mim][Ag(Tf_2N)_2]$ ionic couples, obtained at the ωB97XD/def2-TZVP level in SMD solvent.

	**Tf_2N (trans)**		**N2**		**NO**		**NO2**		**O2**		**O4**
**freq**	**I**	**freq**	**I**	**freq**	**I**	**freq**	**I**	**freq**	**I**	**freq**	**I**
40.7	2.5	31.0	0.0	11.5	0.5	12.5	0.0	5.7	0.0	6.9	0.1
45.9	2.8	39.0	0.6	14.2	1.0	13.9	0.8	12.3	1.5	9.9	0.8
46.8	0.2	48.1	0.6	17.0	1.1	15.3	1.2	15.6	1.3	12.9	0.8
57.2	0.1	75.1	0.3	27.6	0.7	26.7	0.1	23.1	0.4	31.0	0.1
115.2	0.0	125.7	0.3	31.1	0.2	35.7	0.0	23.5	3.2	38.1	1.0
171.7	0.4	158.7	0.0	34.5	1.0	40.5	0.2	31.7	0.3	38.7	1.1
200.4	6.4	195.7	0.6	39.4	0.2	42.4	1.2	33.8	0.2	43.0	0.3
208.6	1.7	202.7	0.0	41.5	0.6	45.7	0.1	40.1	0.8	43.3	0.0
223.7	21.7	205.3	1.0	46.7	1.1	48.2	0.4	41.2	0.2	45.9	0.5
275.2	0.3	279.1	1.0	47.8	1.7	49.8	1.2	44.9	0.3	46.4	1.8
301.5	1.5	286.6	1.2	55.4	0.4	50.5	1.8	46.5	0.1	46.9	1.0
315.3	0.3	311.6	0.9	58.9	1.9	52.9	1.2	49.3	2.1	49.8	2.0
321.9	0.4	327.2	0.9	61.3	1.3	60.4	3.7	49.3	2.3	53.8	1.6
343.8	0.2	333.3	0.3	73.2	5.3	73.0	3.3	62.6	4.6	72.9	0.1

359.9	6.9	352.9	5.0	88.0	5.1	76.8	0.1	75.6	11.7	89.5	3.9
400.8	13.3	409.3	4.6	90.0	0.1	102.9	3.0	87.1	0.4	91.1	4.5
413.7	18.2	441.1	0.1	115.4	0.1	116.3	1.9	112.4	2.5	111.0	32.8
516.7	141.9	511.7	0.2	119.7	0.0	117.3	0.0	118.4	0.0	117.0	1.9
539.1	0.1	536.5	18.4	156.6	24.6	131.9	31.0	153.4	31.6	119.2	10.2
556.9	2.6	555.7	0.0	179.6	3.3	190.7	0.8	171.1	0.1	189.8	0.7
564.6	2.8	565.5	12.0	200.6	5.8	199.4	6.1	178.6	9.9	196.6	24.4
577.0	107.3	577.5	0.0	201.5	6.1	203.6	7.2	202.2	3.8	202.5	21.7
595.0	0.1	585.6	3.6	207.9	1.0	207.1	1.4	202.6	8.1	202.7	0.2
619.3	559.3	603.2	3.7	209.3	2.3	209.7	1.9	208.9	0.0	208.7	1.6
636.3	9.7	656.9	21.1	222.1	0.8	211.2	23.6	212.1	4.3	209.7	2.4
753.0	33.9	754.1	21.1	226.3	30.1	223.7	20.1	223.3	34.0	222.6	22.0
776.2	10.4	776.4	7.4	231.5	21.2	232.8	24.4	223.6	9.8	223.2	22.0
804.6	43.4	801.9	0.0	275.3	1.1	273.7	1.3	277.1	0.3	275.5	0.1
1090.0	830.4	1094.5	4.6	276.3	1.0	276.9	0.1	277.7	1.4	275.9	0.3
1157.2	3.0	1156.7	0.9	295.2	1.0	294.8	0.8	302.0	0.0	303.8	1.4
1166.1	20.9	1167.1	0.9	304.4	1.1	305.2	1.3	302.6	2.0	303.9	1.5
1185.8	215.6	1186.3	0.0	313.0	0.4	311.8	0.4	316.2	0.8	315.4	0.8
1190.4	3.6	1189.9	0.7	321.2	0.7	316.1	0.8	316.2	0.4	315.6	0.7
1190.8	435.7	1195.2	0.6	321.9	0.7	321.1	0.6	322.9	0.7	321.8	0.6
1209.1	1956.5	1205.4	0.6	324.5	1.1	322.4	0.7	323.5	2.3	322.8	0.5
1255.4	193.3	1259.7	0.0	345.8	1.3	342.2	1.7	346.5	0.1	344.3	0.0
1272.3	27.0	1272.4	4.8	350.3	2.9	344.8	0.0	347.7	3.2	344.5	0.0
1367.4	110.9	1364.6	10.1	361.6	3.1	361.1	5.4	360.0	5.5	360.4	5.9
1381.2	792.8	1376.9	10.1	368.7	9.5	369.8	10.1	360.7	1.7	361.6	6.2
			0.0	398.1	9.0	395.0	7.3	408.1	8.3	404.7	0.2
			16.9	406.1	6.8	405.8	7.3	408.7	32.0	405.3	12.5
			6.2	409.2	19.3	407.7	6.3	430.8	0.0	415.9	16.2
			6.2	437.4	11.7	416.6	15.4	441.1	28.5	416.0	15.5
			137.6	515.8	135.9	514.9	128.3	520.2	27.0	520.5	131.0
			137.8	521.3	146.4	522.2	139.3	521.2	265.5	520.6	133.1
			6.7	539.0	0.4	538.8	2.4	539.6	0.0	540.4	0.0
			0.0	539.1	2.6	541.3	0.0	539.6	0.4	540.6	0.1
			0.0	557.7	2.9	557.1	4.3	557.3	1.5	556.9	4.6
			4.2	561.0	2.2	559.3	2.8	557.3	4.9	557.3	3.9
			0.8	565.3	1.0	564.5	0.6	566.0	4.1	565.7	1.4
			0.8	565.9	3.8	565.9	1.7	566.2	2.1	565.7	2.1
			87.3	577.6	90.5	576.6	80.2	578.9	214.9	579.2	116.5
			87.3	578.8	117.3	580.5	142.6	578.9	26.9	579.2	121.8
			0.0	592.0	0.5	591.2	0.1	595.9	0.3	599.5	0.2
			0.3	594.2	1.1	601.1	0.1	595.9	0.3	600.1	0.9
			527.3	617.8	428.0	617.5	172.6	617.4	283.4	617.5	523.4
			527.7	618.6	655.5	619.2	871.5	617.6	791.3	618.2	522.0
			0.0	635.1	13.5	631.3	13.4	634.7	7.4	630.0	18.0
			17.8	665.4	4.0	658.8	0.9	635.1	15.8	631.0	8.2

			0.0	755.4	35.1	754.5	27.0	755.0	15.0	753.6	37.9
			48.3	770.7	38.4	763.2	65.6	755.0	53.5	753.7	15.8
			3.1	779.5	8.4	779.7	3.9	779.3	7.1	779.7	5.3
			3.1	780.4	3.7	780.2	5.2	779.4	9.8	779.9	5.6
			0.0	810.4	45.6	806.6	110.8	810.3	71.2	808.9	45.7
			296.2	815.6	134.2	809.9	31.6	810.3	17.6	809.0	26.7
			879.5	1023.7	902.7	1031.3	868.4	1088.4	1170.4	1088.5	784.3
			879.8	1088.0	734.6	1088.6	812.9	1088.7	365.7	1088.8	787.2
			0.0	1146.4	199.4	1147.8	62.1	1147.6	246.1	1146.5	110.2
			20.8	1150.6	16.3	1150.9	11.0	1147.7	81.1	1148.5	0.9
			421.7	1158.2	409.6	1158.8	328.9	1167.1	529.0	1164.9	149.3
			421.9	1165.6	295.6	1165.3	205.7	1167.3	4.6	1165.1	159.9
			962.1	1195.5	204.8	1196.5	442.6	1195.9	81.8	1195.8	0.8
			0.1	1197.2	386.9	1197.9	220.7	1196.3	59.1	1196.4	50.9
			217.9	1197.5	498.7	1199.0	229.5	1196.8	539.8	1200.8	208.2
			218.0	1199.9	554.1	1201.6	147.2	1196.9	136.7	1201.0	219.6
			0.1	1202.5	404.0	1204.2	124.0	1202.7	1404.9	1202.7	1267.7
			360.1	1207.2	157.5	1205.1	560.1	1203.4	241.0	1203.7	42.5
			973.0	1210.7	978.4	1209.0	436.5	1210.7	2596.4	1210.3	1237.6
			973.4	1212.0	1153.4	1212.5	1852.2	1211.8	4.9	1210.6	1208.1
			169.2	1243.8	171.0	1243.9	155.2	1258.8	582.3	1260.3	373.4
			169.2	1257.9	344.9	1261.0	374.2	1259.3	14.6	1260.7	381.4
			90.9	1266.5	26.3	1265.6	220.4	1267.3	8.2	1264.8	382.6
			0.0	1271.8	29.3	1271.8	30.1	1267.6	13.9	1265.8	3.2
			101.9	1342.9	305.3	1356.7	335.6	1340.6	152.7	1355.2	307.1
			101.9	1387.2	539.9	1374.4	892.4	1342.1	471.7	1355.7	312.3
			1524.1	1396.5	107.1	1393.1	106.3	1388.6	311.3	1369.8	1652.1
			0.0	1413.0	741.5	1409.2	695.0	1388.7	768.5	1375.6	5.3

	**$C4Tf_2N$ (trans)**		**N2-C4**		**NO-C4**		**NO2-C4**		**O2-C4**		**O4-C4**
**freq**	**I**	**freq**	**I**	**freq**	**I**	**freq**	**I**	**freq**	**I**	**freq**	**I**
12.9	0.4	12.8	0.5	9.4	2.1	8.8	1.2	9.2	1.7	7.3	1.7
21.5	1.1	14.5	0.3	17.1	0.8	16.1	0.9	13.1	1.1	14.8	1.7
30.2	4.5	19.5	0.8	19.6	1.5	19.6	2.7	16.1	0.5	21.7	1.5
35.2	1.7	23.1	0.3	23.5	0.6	24.1	1.0	24.0	2.0	25.6	3.8
40.1	3.6	23.6	1.9	25.1	1.1	29.7	3.0	28.4	0.9	30.4	3.2
42.9	0.3	27.0	0.7	26.5	1.8	32.2	0.6	35.8	0.3	36.7	0.7
48.1	0.9	34.5	2.8	35.5	5.9	36.3	2.4	36.8	2.0	38.5	0.4
51.9	1.2	36.0	1.4	38.4	1.9	37.3	1.8	40.6	2.7	39.7	0.4
57.0	1.4	38.3	2.7	40.0	1.2	39.2	0.6	43.3	0.0	46.1	2.5
63.9	2.2	40.6	4.3	43.0	0.4	44.7	2.2	46.0	2.8	48.2	4.2
69.2	7.6	44.5	0.5	45.7	2.8	45.8	1.1	47.5	1.5	49.1	0.4
95.5	11.3	47.6	2.6	51.0	5.3	49.6	0.7	49.4	3.6	52.7	5.6
117.6	0.9	52.1	0.3	54.2	2.4	50.9	2.0	53.6	1.7	53.7	3.7

120.3	0.2	53.5	0.2	58.6	5.2	56.7	2.4	57.3	2.1	56.7	1.6
126.4	0.3	59.8	11.2	61.9	2.9	60.1	2.9	62.3	0.9	59.0	1.7
143.2	0.1	62.2	0.3	62.8	1.2	63.2	0.8	64.2	5.2	66.4	1.5
168.1	0.3	65.9	1.0	65.8	4.2	65.8	0.3	71.4	1.0	69.9	0.5
203.3	6.7	68.2	1.4	71.6	3.1	71.8	1.5	74.9	3.5	73.9	3.0
210.5	0.9	71.1	5.1	75.3	0.5	73.3	5.5	77.3	1.4	74.8	1.2
226.4	13.6	74.1	0.9	76.9	0.6	75.3	3.9	83.2	6.6	76.5	1.1
227.8	9.6	82.3	2.4	84.6	1.7	76.3	0.7	90.6	5.5	83.3	3.8
252.4	3.8	85.1	0.3	89.4	1.4	85.1	6.2	95.6	9.5	90.0	0.9
270.4	0.8	92.5	3.5	92.8	2.5	91.2	5.1	100.5	4.3	94.9	5.4
277.5	0.4	97.7	0.4	102.8	0.4	97.9	2.1	109.6	5.2	103.3	3.3
301.4	0.8	113.9	0.0	121.0	0.2	104.3	0.9	112.0	6.0	109.3	8.1
303.0	0.7	120.3	0.4	122.7	0.2	113.2	1.1	126.8	2.6	117.2	0.8
317.5	0.6	121.6	0.2	128.3	0.3	123.4	7.6	130.6	0.2	119.6	1.4
322.7	0.5	138.7	0.1	140.8	14.6	124.4	4.6	151.8	10.5	123.5	22.3
345.1	0.6	155.9	20.4	147.2	12.6	134.2	7.2	158.7	3.2	153.7	2.1
359.0	0.9	173.3	0.7	156.6	2.4	135.3	10.5	161.1	1.7	156.4	0.5
363.8	6.6	199.1	8.0	164.8	3.7	186.8	4.3	188.9	2.3	183.7	8.8
402.4	14.8	200.1	1.3	201.2	7.1	194.8	14.4	197.0	0.3	189.6	19.4
413.0	12.8	202.9	3.8	203.7	5.8	201.2	5.2	201.6	2.2	200.6	3.4
420.4	1.5	206.6	2.1	209.9	3.6	210.1	4.6	205.5	1.5	207.1	4.1
448.3	0.3	211.3	0.9	210.8	1.5	211.4	0.4	206.6	6.9	209.2	9.6
517.3	138.1	217.8	2.6	213.0	5.7	220.0	8.8	219.8	16.2	216.3	10.1
539.3	0.3	228.3	28.6	216.9	3.4	223.9	2.3	223.7	10.5	219.5	1.9
558.0	2.4	229.4	17.3	222.4	16.4	225.2	10.9	240.0	1.6	224.1	22.2
565.2	2.1	233.6	6.4	231.3	21.6	229.5	25.0	242.5	1.1	235.3	2.3
577.4	101.2	249.8	0.0	247.8	0.1	245.3	4.9	243.9	0.2	253.9	2.7
595.5	0.0	266.1	1.8	259.0	1.9	275.1	1.3	278.4	1.5	276.3	0.1
620.5	538.2	272.8	1.0	274.9	1.2	279.9	0.7	280.4	5.3	278.8	1.8
637.2	16.4	276.5	2.1	277.1	1.4	281.0	0.9	280.6	2.4	286.2	0.8
645.1	36.1	287.7	2.3	294.8	1.0	294.8	1.4	287.4	4.5	303.5	1.2
648.3	4.4	293.3	0.9	301.1	1.3	297.3	0.3	293.7	1.0	308.8	2.0
685.1	24.8	295.5	0.9	307.0	1.0	310.5	2.2	302.9	2.1	312.7	1.1
747.4	7.4	313.1	0.6	313.9	0.5	314.3	0.6	310.7	0.7	314.0	0.8
755.7	38.6	314.3	0.6	320.7	1.3	315.1	1.4	317.7	0.7	315.6	0.8
770.3	61.0	320.3	0.6	322.2	0.8	322.3	1.1	329.2	0.5	322.4	1.2
777.1	8.8	322.7	1.0	327.1	0.7	328.8	2.8	330.1	1.5	328.6	2.0
779.2	12.6	329.4	0.4	333.4	0.9	341.4	0.8	333.7	0.8	341.2	0.9
806.4	5.3	344.0	0.3	344.5	1.9	343.7	2.0	341.7	5.6	343.7	0.1
808.8	57.5	345.0	4.4	352.6	1.4	357.8	2.5	352.7	0.6	350.2	1.3
887.3	41.5	366.5	6.6	364.6	4.4	367.3	8.4	353.8	2.6	355.8	1.1
901.3	5.8	368.9	10.2	368.2	8.6	369.3	0.2	369.1	7.6	359.8	4.7
906.5	1.1	396.0	3.9	396.5	8.8	395.6	7.2	403.8	12.6	405.6	6.4
968.0	1.7	399.2	13.0	405.8	5.5	405.5	5.4	413.9	2.9	412.7	8.9
1000.0	1.6	404.7	4.6	408.4	16.6	412.2	7.4	426.8	8.9	416.8	17.5

1057.6	3.5	406.2	7.0	425.8	0.7	417.6	25.4	434.4	27.9	425.6	33.4
1062.1	2.6	407.9	2.0	434.2	6.9	422.3	0.4	443.9	18.7	429.9	1.0
1087.2	790.7	466.8	2.0	459.3	0.4	444.9	1.7	464.5	0.5	454.6	0.6
1098.8	0.8	515.0	68.9	514.9	138.9	516.0	133.7	514.5	195.3	517.5	116.8
1115.7	10.4	515.9	203.7	520.1	150.9	519.3	128.8	517.3	150.6	520.8	155.6
1129.0	5.2	539.3	5.5	538.5	0.7	539.1	2.5	536.0	15.3	541.4	0.8
1139.6	3.0	539.5	1.2	538.8	2.7	542.1	1.0	540.9	0.2	542.5	1.4
1156.1	5.9	561.1	0.8	559.2	3.7	558.6	6.1	555.8	6.8	557.2	6.3
1157.1	4.8	561.6	3.2	561.1	2.4	561.1	3.2	558.1	4.8	557.3	5.4
1163.1	1.8	565.4	0.8	566.2	1.4	566.8	1.0	566.8	3.0	566.7	0.9
1167.4	54.1	566.1	0.5	567.8	4.0	568.3	1.9	568.7	4.4	566.9	3.7
1184.7	491.1	576.9	53.4	577.1	85.5	577.8	76.3	577.7	45.7	579.3	53.8
1189.9	84.8	577.2	122.1	579.1	103.4	580.6	117.0	578.2	187.4	580.0	172.3
1193.7	91.6	591.9	0.3	591.7	0.5	593.3	0.2	586.4	58.5	591.0	6.6
1207.7	89.7	592.3	0.2	595.1	2.3	594.6	2.2	598.9	7.4	598.6	6.1
1210.2	1859.7	616.7	149.0	615.3	376.9	613.4	487.0	608.3	207.8	610.9	424.9
1237.8	2.1	619.0	909.5	619.2	632.0	622.0	551.3	619.3	500.3	617.3	538.1
1253.2	167.5	640.3	29.6	633.6	112.6	640.5	42.7	634.2	15.5	630.6	15.4
1272.5	27.2	656.2	6.8	647.8	2.3	642.3	59.2	646.4	5.7	644.9	86.4
1295.9	0.5	665.2	4.3	660.3	5.4	648.2	3.2	654.6	93.5	651.3	4.1
1304.1	0.5	665.8	12.3	663.3	5.7	655.6	2.6	657.2	158.9	655.3	27.4
1330.7	2.7	686.5	10.4	681.3	23.3	684.3	8.7	687.9	20.0	688.5	17.2
1357.0	3.0	749.6	11.2	752.4	10.7	747.6	14.3	750.4	18.7	752.4	7.8
1367.4	78.5	760.6	8.6	753.6	51.2	748.0	79.6	754.5	91.8	754.5	31.7
1382.3	859.5	770.7	8.4	769.6	33.7	756.9	30.0	756.0	24.6	754.7	11.4
1385.3	3.9	772.7	57.8	770.7	22.0	762.6	76.1	761.3	11.8	777.1	7.7
1399.0	4.4	780.2	2.1	780.6	8.0	781.3	4.8	774.4	56.6	780.3	4.7
1417.4	14.6	780.5	3.8	780.9	3.4	781.9	3.2	780.8	6.2	780.8	5.4
1421.2	5.4	809.6	53.9	799.2	87.2	788.8	8.8	781.4	5.5	790.3	107.9
1446.1	5.6	812.8	51.2	811.0	52.0	807.5	109.7	808.7	3.0	808.4	25.7
1467.8	11.7	818.1	7.9	814.0	2.3	811.8	25.1	809.8	93.3	809.7	31.4
1474.2	20.7	820.5	262.1	815.7	136.4	813.3	12.2	811.4	40.5	813.7	4.8
1477.0	2.0	887.2	58.4	889.6	45.2	873.6	26.6	903.9	49.4	901.5	11.5
1480.2	5.4	926.9	1.4	920.0	0.3	902.9	2.1	907.4	2.2	912.3	5.6
1481.9	8.3	936.4	3.5	930.5	2.5	921.4	31.9	932.1	2.2	922.3	46.1
1494.2	7.7	956.3	0.9	958.0	0.9	967.1	0.5	959.8	0.9	974.4	1.2
1500.5	16.7	1023.9	1269.2	1022.9	865.7	999.3	2.8	1039.4	2.1	1003.4	1.0
1505.1	18.3	1024.8	447.1	1042.3	1.5	1025.1	858.4	1056.6	6.1	1063.7	0.7
1513.0	15.2	1040.7	2.9	1064.3	1.9	1056.0	6.8	1066.6	5.9	1065.7	3.6
1635.7	29.2	1062.5	1.2	1069.5	2.6	1064.7	6.5	1087.6	818.2	1089.5	664.6
1643.4	107.1	1071.9	2.3	1086.8	25.6	1088.3	789.2	1088.2	249.1	1092.4	824.8
3045.5	37.6	1085.9	1.7	1087.7	691.0	1100.7	1.0	1088.7	7.8	1097.9	0.1
3047.9	40.5	1119.1	7.4	1122.4	9.7	1114.2	7.3	1116.7	11.2	1121.2	11.0
3051.1	23.6	1142.7	5.2	1139.7	19.1	1133.1	8.1	1129.0	1.4	1137.0	8.1
3089.1	22.7	1144.8	2.8	1146.3	177.3	1137.4	2.7	1144.5	9.2	1146.6	145.6

3094.1	20.9	1149.3	55.7	1147.4	20.6	1148.2	57.4	1146.9	32.9	1149.4	8.7
3098.0	33.7	1150.0	9.1	1149.8	25.5	1149.8	22.3	1147.2	712.6	1150.1	47.8
3109.3	15.0	1157.5	265.5	1158.4	450.1	1157.5	460.1	1164.4	13.1	1161.4	7.3
3117.5	80.1	1159.4	562.6	1163.0	0.1	1159.3	2.7	1168.6	120.8	1165.8	34.7
3124.9	62.2	1162.6	11.4	1166.6	12.3	1167.1	143.6	1169.2	20.6	1166.2	78.5
3167.5	11.6	1170.0	0.4	1168.1	359.4	1173.5	5.8	1170.0	279.1	1168.7	125.7
3180.3	3.6	1192.2	589.0	1189.4	222.5	1191.5	464.9	1192.8	223.3	1194.3	244.7
3196.9	2.5	1197.0	121.5	1194.2	519.4	1195.0	237.9	1197.7	282.1	1194.5	157.2
3303.6	19.1	1198.8	580.5	1197.4	189.7	1203.0	46.3	1203.3	730.0	1200.2	146.3
3315.5	49.1	1202.2	45.6	1198.0	311.0	1203.3	262.8	1204.0	125.4	1203.1	555.3
3321.7	1.1	1205.1	388.9	1204.5	1055.4	1204.3	216.9	1204.3	514.8	1206.7	807.7
		1208.2	170.3	1209.3	65.5	1206.8	761.9	1208.3	780.6	1207.5	479.8
		1210.4	199.6	1210.6	123.4	1210.4	456.0	1210.6	112.9	1211.0	37.1
		1214.7	227.4	1214.3	1084.8	1212.6	487.4	1213.2	604.8	1212.5	345.0
		1216.0	1505.5	1216.3	722.5	1215.5	1172.2	1214.7	1406.1	1215.2	1737.0
		1242.9	170.6	1243.6	182.3	1234.8	6.9	1248.9	3.7	1260.2	4.7
		1243.3	165.6	1248.5	1.7	1242.6	171.3	1258.0	215.3	1262.6	328.6
		1249.5	2.2	1254.8	368.0	1261.0	346.6	1259.7	433.8	1264.2	301.2
		1270.2	87.2	1266.9	2.2	1268.1	175.9	1268.5	129.5	1266.9	141.4
		1272.3	6.8	1271.1	35.3	1270.2	27.2	1268.6	131.0	1272.1	409.8
		1294.4	2.9	1300.6	0.6	1295.5	0.6	1294.2	3.0	1299.7	1.2
		1310.7	2.4	1313.9	0.3	1306.3	1.9	1309.2	2.0	1307.7	0.8
		1331.3	1.6	1332.7	2.5	1332.2	0.6	1331.1	3.3	1341.9	1.7
		1342.2	2.9	1343.4	269.4	1350.8	422.7	1337.0	327.7	1353.2	263.4
		1385.4	8.8	1345.3	50.1	1359.1	2.9	1338.8	5.4	1356.4	433.7
		1394.2	99.6	1392.4	189.3	1376.3	758.6	1349.1	392.1	1367.3	1247.4
		1396.3	167.8	1392.9	299.8	1385.9	7.6	1380.6	483.5	1373.8	59.7
		1398.2	3.0	1396.2	124.6	1393.0	91.9	1383.0	703.3	1375.9	369.1
		1412.4	1366.9	1402.0	4.8	1399.8	5.2	1386.3	3.3	1390.9	5.9
		1414.6	70.2	1412.6	763.5	1409.5	722.4	1396.2	2.9	1398.2	2.9
		1415.7	17.2	1416.7	4.4	1417.5	14.2	1414.1	5.2	1422.9	9.2
		1424.3	53.2	1426.7	16.0	1421.7	2.1	1420.1	13.5	1434.4	11.1
		1444.7	6.8	1442.1	13.7	1443.0	10.6	1444.5	4.1	1440.2	5.3
		1467.0	5.8	1472.0	7.2	1470.2	13.0	1469.7	5.3	1468.7	7.8
		1476.9	5.8	1475.5	7.0	1474.8	1.7	1476.0	3.3	1475.2	21.4
		1477.3	1.7	1478.7	10.1	1478.3	17.2	1478.2	8.4	1479.6	2.0
		1479.5	21.8	1480.6	13.1	1480.5	15.4	1485.0	0.0	1482.4	7.9
		1487.5	0.4	1487.4	5.3	1483.3	1.8	1487.0	12.9	1484.7	6.8
		1489.2	12.0	1488.6	7.0	1494.2	3.5	1491.8	22.4	1498.5	8.2
		1500.5	10.5	1501.2	7.4	1500.9	21.6	1500.7	10.9	1504.5	21.6
		1512.1	14.7	1511.4	15.2	1504.7	12.3	1512.6	25.2	1510.4	21.8
		1513.5	30.1	1512.1	26.1	1507.2	11.9	1515.4	19.5	1513.7	18.7
		1634.5	37.4	1626.4	13.7	1632.0	62.7	1632.2	39.2	1632.6	13.3
		1646.4	88.3	1641.9	99.4	1640.8	59.1	1641.9	92.0	1645.3	107.7
		3043.7	48.9	3043.7	47.2	3044.8	38.1	3044.0	39.0	3048.4	55.8

		3045.3	9.0	3045.2	8.3	3052.0	31.8	3048.5	5.9	3049.9	23.7
		3054.1	59.8	3053.5	61.8	3059.9	25.2	3056.1	82.3	3058.8	20.3
		3076.8	2.9	3076.7	2.8	3093.4	11.2	3079.4	0.9	3091.1	28.3
		3095.0	15.7	3093.1	13.7	3097.3	14.0	3097.2	20.8	3091.9	15.3
		3100.7	24.1	3099.9	24.9	3102.4	14.6	3097.8	14.1	3105.2	36.0
		3106.9	17.0	3104.2	12.4	3113.8	16.6	3100.6	32.1	3106.7	7.7
		3116.5	99.8	3116.1	100.8	3118.1	82.9	3116.5	105.4	3122.7	54.2
		3122.6	53.0	3123.4	52.2	3122.9	61.5	3123.8	53.4	3126.8	69.7
		3162.4	11.5	3162.2	8.1	3161.8	10.7	3156.5	15.7	3164.0	7.5
		3185.3	3.1	3183.0	2.7	3190.2	0.8	3184.5	3.4	3179.4	2.8
		3195.9	2.6	3197.1	1.8	3196.0	0.6	3203.0	1.7	3197.8	1.5
		3309.4	45.1	3308.8	25.3	3305.8	48.9	3272.6	220.3	3306.5	30.7
		3320.1	33.2	3324.9	29.1	3307.9	34.6	3300.8	42.1	3313.8	55.6
		3336.6	15.1	3336.1	43.0	3324.6	11.9	3317.6	15.2	3325.4	24.4

Table S2. Ag-N and Ag-O bond lengths (Å, averaged) of the minimum energy structures obtained at the ωB97XD/def2-TZVP level in SMD solvent.

	Ag-N	Ag-O		Ag-N	Ag-O
**N2**	2.209	-	**N2-C4**	2.217	-
**NO**	2.222	2.224	**NO-C4**		
**O2**	-	2.242	**O2-C4**	-	2.236
**NO2**	2.252	2.408	**NO2-C4**	2.269	2.442
**O4**	-	2.436	**O4-C4**	-	2.449

Cartesian Coordinates

Coordinates of the minimum energy structures (in SMD solvent) of the species considered in this study.

15
Tf_2N^- (trans)

S	1.136232000	0.833084000	0.046649000
O	1.581270000	1.851698000	0.948298000
N	0.000086000	-0.000337000	0.742417000
S	-1.136386000	-0.833298000	0.046658000
O	0.917422000	1.182554000	-1.324743000
O	-0.917679000	-1.182876000	-1.324723000
O	-1.581751000	-1.851745000	0.948337000
C	-2.546543000	0.368799000	0.002104000
F	-2.851532000	0.787851000	1.225089000
F	-2.246760000	1.425621000	-0.744616000
F	-3.615567000	-0.227840000	-0.518964000
C	2.546719000	-0.368612000	0.002122000
F	3.615564000	0.228287000	-0.519018000
F	2.247170000	-1.425540000	-0.744548000
F	2.851871000	-0.787532000	1.225108000

15
Tf_2N^- (cis)

S	1.473709000	-0.844706000	0.312634000
O	1.744678000	-1.640022000	-0.847305000
N	-0.038248000	-0.718772000	0.698061000
S	-1.289879000	-0.840324000	-0.245408000
O	2.230523000	-1.085793000	1.503272000
O	-1.035538000	-0.733529000	-1.651631000
O	-2.172060000	-1.874276000	0.206045000
C	-2.173814000	0.724964000	0.200736000
F	-2.458561000	0.762093000	1.496760000
F	-1.441376000	1.790332000	-0.103900000
F	-3.312578000	0.785942000	-0.484689000
C	1.981999000	0.871162000	-0.175081000
F	3.294834000	0.894509000	-0.392331000
F	1.699651000	1.738576000	0.791972000
F	1.366531000	1.260113000	-1.285479000

40
[C_4mim][Tf_2N] (trans)

C	1.242287000	0.862149000	2.533996000
C	2.109408000	1.109088000	1.526003000
C	1.621729000	-1.025531000	1.489823000
N	0.956159000	-0.478851000	2.496691000
H	0.797124000	1.517371000	3.261187000
H	2.579747000	2.021736000	1.205678000
H	1.576448000	-2.059580000	1.195703000
N	2.330924000	-0.084677000	0.885520000
C	-0.011893000	-1.159781000	3.338333000
H	-1.018480000	-0.891091000	3.022046000
H	0.132167000	-2.232845000	3.245807000
H	0.142489000	-0.859539000	4.372363000
C	3.208137000	-0.285489000	-0.264573000
H	2.975969000	-1.266583000	-0.675736000
H	2.933763000	0.462792000	-1.006640000
C	4.675982000	-0.188349000	0.122774000
H	4.874993000	0.804566000	0.537026000
H	4.873361000	-0.914432000	0.915564000
C	5.610871000	-0.449624000	-1.054754000
H	5.411404000	-1.446766000	-1.459157000
H	6.634754000	-0.470340000	-0.674059000
C	5.512540000	0.584572000	-2.169268000
H	6.266500000	0.398915000	-2.936612000
H	4.537074000	0.566353000	-2.659765000
H	5.673314000	1.593888000	-1.780813000
S	-1.999221000	1.157365000	0.324524000
O	-1.877948000	1.509809000	1.706885000
N	-0.954626000	0.027422000	-0.003148000
S	-1.029674000	-1.022373000	-1.170891000
O	-3.310092000	0.983030000	-0.222636000
O	-1.874612000	-0.684729000	-2.275189000

O	0.293348000	-1.499573000	-1.442896000
C	-1.863325000	-2.471887000	-0.370202000
F	-1.155713000	-2.910010000	0.667804000
F	-3.077936000	-2.149087000	0.054942000
F	-1.968919000	-3.459036000	-1.253756000
C	-1.312944000	2.626276000	-0.575401000
F	-2.088988000	3.680343000	-0.342439000
F	-1.276840000	2.405988000	-1.883832000
F	-0.082475000	2.908644000	-0.160928000

40

[C_4mim][Tf_2N] (cis)

C	3.144475000	-2.241743000	-0.083184000
C	3.084473000	-1.065323000	0.581374000
C	2.180701000	-0.772346000	-1.392604000
N	2.575892000	-2.034328000	-1.315851000
H	3.529319000	-3.202523000	0.210084000
H	3.408047000	-0.796534000	1.571347000
H	1.677693000	-0.319998000	-2.229831000
N	2.477833000	-0.161614000	-0.255567000
C	2.383138000	-3.046852000	-2.339797000
H	1.723335000	-3.823438000	-1.957663000
H	1.932286000	-2.581761000	-3.212388000
H	3.345821000	-3.476927000	-2.609005000
C	2.170939000	1.230802000	0.061232000
H	1.501248000	1.592470000	-0.717595000
H	1.622002000	1.229406000	1.001633000
C	3.425745000	2.085686000	0.140166000
H	4.073863000	1.706588000	0.935995000
H	3.974575000	1.985222000	-0.799551000
C	3.107585000	3.557103000	0.391415000
H	2.478402000	3.931726000	-0.421831000
H	4.043357000	4.118840000	0.342144000
C	2.429857000	3.828597000	1.728736000
H	2.312005000	4.901618000	1.891365000
H	1.435507000	3.380286000	1.781877000
H	3.022357000	3.428730000	2.556142000
S	-1.491163000	0.444598000	-1.490587000
O	-2.575180000	-0.208815000	-2.157606000
N	-0.738064000	-0.412548000	-0.420402000
S	-1.243410000	-1.676771000	0.363232000
O	-0.516128000	1.134431000	-2.283980000
O	-2.650287000	-1.935612000	0.305361000
O	-0.341293000	-2.774226000	0.178226000
C	-0.927736000	-1.132584000	2.105860000
F	0.361129000	-0.875980000	2.302243000
F	-1.624973000	-0.042239000	2.399297000
F	-1.294270000	-2.107014000	2.931929000
C	-2.287509000	1.802578000	-0.512854000
F	-2.828419000	2.686008000	-1.345515000
F	-1.387179000	2.427439000	0.240281000
F	-3.242196000	1.322760000	0.273945000

31

N2 (trans,trans)

Ag	-0.000007000	0.003220000	0.004236000
N	2.208656000	-0.000645000	0.000093000
S	2.946727000	-0.222306000	-1.409332000
S	2.952765000	0.218611000	1.406692000
O	4.300116000	-0.245275000	1.440318000
O	2.043172000	-0.152062000	2.443083000
O	4.295506000	0.237029000	-1.447825000
O	2.034587000	0.151883000	-2.442213000
C	3.044718000	-2.072291000	-1.526644000
C	3.057788000	2.068265000	1.523066000
F	1.846268000	2.604698000	1.471041000
F	3.789341000	2.561360000	0.535858000
F	3.621148000	2.388012000	2.680588000
F	1.831466000	-2.604366000	-1.470303000
F	3.778186000	-2.568361000	-0.542349000
F	3.602588000	-2.393744000	-2.686351000
S	-2.949040000	-1.407372000	0.219173000
O	-2.036869000	-2.440965000	-0.152961000
N	-2.208650000	0.001428000	0.001345000
S	-2.950447000	1.409124000	-0.218637000
O	-4.296378000	-1.443950000	-0.244532000

O	-4.299221000	1.443664000	0.241024000
O	-2.040881000	2.443979000	0.156371000
C	-3.049262000	1.527974000	-2.068504000
F	-1.836023000	1.475612000	-2.601016000
F	-3.780028000	0.542057000	-2.565327000
F	-3.610559000	2.686391000	-2.388639000
C	-3.053249000	-1.526277000	2.068694000
F	-3.612809000	-2.685971000	2.387240000
F	-3.787832000	-0.542003000	2.563160000
F	-1.841745000	-1.471039000	2.604844000

31
N2 (cis,trans)

Ag	-0.042804000	-0.020136000	-0.376476000
N	2.152692000	-0.247582000	-0.578068000
S	2.891943000	1.000397000	-1.261734000
S	2.820774000	-1.679659000	-0.282724000
O	4.227388000	-1.723803000	-0.513763000
O	1.979297000	-2.712445000	-0.794794000
O	1.869273000	1.908892000	-1.676498000
O	3.935646000	0.642187000	-2.163330000
C	3.730108000	1.883692000	0.139146000
C	2.624291000	-1.806960000	1.561121000
F	1.343710000	-1.813911000	1.905905000
F	3.228126000	-0.800756000	2.174230000
F	3.178291000	-2.946582000	1.952778000
F	4.738801000	1.171103000	0.616185000
F	2.865152000	2.121442000	1.115621000
F	4.197755000	3.039642000	-0.313132000
S	-3.176670000	-0.737453000	-1.023147000
O	-2.458800000	-0.969853000	-2.235071000
N	-2.225290000	0.110901000	-0.045539000
S	-2.737441000	1.042603000	1.158392000
O	-4.528115000	-0.287119000	-1.068813000
O	-4.047584000	0.735054000	1.627691000
O	-1.661504000	1.186496000	2.086230000
C	-2.874073000	2.706597000	0.347460000
F	-1.699739000	3.078688000	-0.143421000
F	-3.761939000	2.680814000	-0.634317000
F	-3.260444000	3.591232000	1.257202000
C	-3.231666000	-2.396840000	-0.192713000
F	-3.965663000	-3.215958000	-0.934289000
F	-3.773427000	-2.302761000	1.011944000
F	-2.010010000	-2.899088000	-0.077183000

31
N2 (cis,cis)

Ag	0.000120000	-0.000093000	-0.540135000
N	-2.188810000	-0.392025000	-0.490752000
S	-2.593920000	-1.736918000	0.283291000
S	-3.184408000	0.502182000	-1.382600000
O	-4.552584000	0.107244000	-1.301940000
O	-2.587129000	0.753624000	-2.654157000
O	-1.374752000	-2.401105000	0.623478000
O	-3.656156000	-2.466909000	-0.324356000
C	-3.258003000	-1.164245000	1.918906000
C	-3.095493000	2.139062000	-0.506912000
F	-1.867927000	2.637109000	-0.552408000
F	-3.464564000	2.021558000	0.759352000
F	-3.920852000	2.976363000	-1.120995000
F	-4.413757000	-0.535639000	1.771551000
F	-2.391433000	-0.349666000	2.505629000
F	-3.443272000	-2.225745000	2.692502000
S	2.594195000	1.737047000	0.282847000
O	1.375103000	2.401602000	0.622569000
N	2.188999000	0.391911000	-0.490709000
S	3.184492000	-0.502554000	-1.382444000
O	3.656684000	2.466612000	-0.324871000
O	2.587276000	-0.753998000	-2.654031000
O	4.552756000	-0.107925000	-1.301725000
C	3.095163000	-2.139289000	-0.506509000
F	3.463622000	-2.021520000	0.759905000
F	1.867621000	-2.637346000	-0.552503000
F	3.920816000	-2.976710000	-1.120035000
C	3.257833000	1.164824000	1.918801000
F	3.443027000	2.226559000	2.692099000

F 4.413553000 0.536041000 1.771955000
F 2.391024000 0.350527000 2.505570000

31
NO
Ag 0.683918000 -0.349435000 -0.432206000
N 2.851221000 -0.167418000 0.023516000
S 3.570710000 1.201260000 -0.412619000
S 3.577728000 -1.388390000 0.773323000
O 2.571355000 -2.148334000 1.442723000
O 4.776963000 -1.033651000 1.456794000
O 2.812808000 1.771493000 -1.480244000
O 4.989996000 1.115976000 -0.512385000
C 3.237149000 2.315312000 1.034404000
C 4.108681000 -2.470687000 -0.638226000
F 4.963663000 -1.836860000 -1.425603000
F 3.056083000 -2.840771000 -1.356238000
F 4.695229000 -3.553167000 -0.144972000
F 3.784285000 1.831756000 2.138675000
F 1.931239000 2.449280000 1.223258000
F 3.759324000 3.507354000 0.778561000
S -2.510353000 0.385292000 -0.148117000
O -1.490317000 -0.329353000 -0.897583000
N -3.887218000 -0.029863000 -0.738594000
S -5.288126000 0.007196000 -0.005428000
O -2.366762000 0.426992000 1.272132000
O -5.345591000 0.816113000 1.172289000
O -6.313600000 0.139343000 -0.990672000
C -5.449094000 -1.736753000 0.601338000
F -5.406032000 -2.592728000 -0.411161000
F -4.472345000 -2.030208000 1.450649000
F -6.613479000 -1.871593000 1.226076000
C -2.297264000 2.140612000 -0.710512000
F -1.123089000 2.595504000 -0.287099000
F -3.259111000 2.899319000 -0.206554000
F -2.334340000 2.215353000 -2.032455000

31
O2
Ag 0.000075000 -0.081072000 0.000174000
N -4.479169000 -0.352368000 -0.780856000
S -3.256149000 0.480187000 -0.302451000
S -5.883319000 -0.486536000 -0.067773000
O -6.864459000 -0.814459000 -1.052483000
O -6.180162000 0.521312000 0.902171000
O -2.065898000 -0.128127000 -0.870326000
O -3.197160000 0.866287000 1.071951000
C -3.400026000 2.066256000 -1.249755000
C -5.669848000 -2.044768000 0.912534000
F -4.703251000 -1.903524000 1.811703000
F -5.368241000 -3.063864000 0.118790000
F -6.805284000 -2.317232000 1.545385000
F -4.501036000 2.711999000 -0.894731000
F -3.436431000 1.833002000 -2.552906000
F -2.345672000 2.827055000 -0.978056000
S 3.256212000 0.480243000 0.302579000
O 2.066002000 -0.127850000 0.870779000
N 4.479243000 -0.352271000 0.780960000
S 5.883250000 -0.486783000 0.067727000
O 3.197026000 0.866027000 -1.071906000
O 6.180173000 0.520962000 -0.902302000
O 6.864440000 -0.814854000 1.052338000
C 5.669415000 -2.045000000 -0.912520000
F 5.367756000 -3.064054000 -0.118745000
F 4.702714000 -1.903616000 -1.811568000
F 6.804727000 -2.317626000 -1.545513000
C 3.400360000 2.066528000 1.249468000
F 2.345985000 2.827310000 0.977828000
F 4.501327000 2.712121000 0.894001000
F 3.437070000 1.833610000 2.552664000

31
NO2
Ag -0.051077000 -0.001195000 0.000092000
N -2.303180000 -0.000105000 -0.000194000
S -3.039803000 1.420947000 -0.082900000

S	-3.040808000	-1.420662000	0.082152000
O	-2.133854000	-2.414132000	-0.397502000
O	-4.393536000	-1.416701000	-0.369169000
O	-2.132392000	2.413812000	0.397126000
O	-4.392737000	1.417906000	0.367815000
C	-3.128097000	1.722557000	-1.912008000
C	-3.130091000	-1.722271000	1.911209000
F	-3.857405000	-0.789719000	2.507718000
F	-1.914318000	-1.723250000	2.441985000
F	-3.690858000	-2.906360000	2.119699000
F	-3.855955000	0.790578000	-2.508753000
F	-1.912118000	1.722506000	-2.442322000
F	-3.687800000	2.907101000	-2.120770000
S	3.199981000	1.409282000	0.093299000
O	1.824493000	1.469852000	-0.340866000
N	3.878333000	0.000585000	0.000618000
S	3.201243000	-1.408670000	-0.092534000
O	4.088033000	2.406983000	-0.404650000
O	4.089819000	-2.405636000	0.405954000
O	1.825526000	-1.470471000	0.340780000
C	3.112256000	-1.714771000	-1.918412000
F	2.356680000	-0.794293000	-2.505489000
F	4.322871000	-1.682480000	-2.454304000
F	2.576361000	-2.909075000	-2.131855000
C	3.109618000	1.715290000	1.919128000
F	2.572192000	2.908955000	2.132278000
F	4.319969000	1.684423000	2.455699000
F	2.354802000	0.793914000	2.505786000

31
O4

Ag	-0.008975000	-0.032487000	-0.007638000
N	-3.953783000	0.054177000	-0.027131000
S	-3.253846000	0.188611000	-1.421647000
S	-3.302797000	-0.144183000	1.383845000
O	-4.195933000	0.322010000	2.393206000
O	-1.917159000	0.240713000	1.493667000
O	-4.157168000	-0.202456000	-2.453332000
O	-1.903956000	-0.316234000	-1.496760000
C	-3.065779000	2.022290000	-1.622050000
C	-3.276837000	-1.986908000	1.583315000
F	-2.515826000	-2.544280000	0.649249000
F	-4.501757000	-2.482565000	1.490271000
F	-2.779371000	-2.286988000	2.776223000
F	-2.304838000	2.520652000	-0.654859000
F	-4.248065000	2.618093000	-1.585465000
F	-2.491754000	2.274020000	-2.791538000
S	3.259872000	1.401223000	0.148137000
O	1.888204000	1.465669000	-0.294258000
N	3.950078000	0.000615000	0.022596000
S	3.286832000	-1.412936000	-0.104994000
O	4.146156000	2.417474000	-0.315869000
O	4.188133000	-2.411196000	0.369168000
O	1.913194000	-1.499829000	0.326575000
C	3.204068000	-1.676257000	-1.939050000
F	2.444469000	-0.748107000	-2.508265000
F	4.416502000	-1.622719000	-2.469892000
F	2.676339000	-2.868451000	-2.185250000
C	3.159687000	1.658637000	1.981760000
F	2.615842000	2.844057000	2.225715000
F	4.368700000	1.618652000	2.521689000
F	2.407337000	0.720317000	2.543481000

56
N2-C4

Ag	-0.726688000	-0.224757000	0.144521000
N	1.137607000	-1.420021000	0.250015000
S	1.521961000	-2.086525000	1.663257000
S	1.918993000	-1.673711000	-1.126994000
O	3.305128000	-1.971037000	-0.976174000
O	1.506061000	-0.658847000	-2.045110000
O	2.466183000	-3.149534000	1.569390000
O	0.323166000	-2.252801000	2.418147000
C	2.416893000	-0.708171000	2.522329000
C	1.133325000	-3.217927000	-1.797323000
F	-0.172428000	-3.036750000	-1.940410000
F	1.340888000	-4.249226000	-0.996487000

F 1.666468000 -3.475510000 -2.984008000
F 1.623307000 0.346635000 2.663285000
F 3.494461000 -0.347746000 1.839473000
F 2.788593000 -1.128529000 3.722173000
S -3.152018000 1.939304000 -0.477791000
O -4.511937000 2.112190000 -0.865040000
N -2.814917000 0.514149000 0.173286000
S -3.895701000 -0.459304000 0.862980000
O -2.103970000 2.252545000 -1.398410000
O -3.196754000 -1.305098000 1.775521000
O -5.097094000 0.192308000 1.265475000
C -4.377644000 -1.579887000 -0.536763000
F -4.921030000 -0.888208000 -1.526444000
F -3.316588000 -2.228792000 -0.997231000
F -5.260171000 -2.460707000 -0.087234000
C -2.879154000 3.110709000 0.935873000
F -3.043162000 4.350994000 0.496079000
F -1.643296000 2.982765000 1.406803000
F -3.736319000 2.880338000 1.915654000
C 2.740565000 2.057043000 -2.414664000
H 3.251932000 1.893795000 -3.347879000
C 2.299519000 2.160930000 -0.271972000
C 1.184262000 2.525514000 -0.946533000
H 0.215496000 2.840193000 -0.601800000
H 2.494896000 2.103417000 0.783111000
N 3.260542000 1.873422000 -1.209738000
N 1.484707000 2.456769000 -2.283457000
C 0.555760000 2.701004000 -3.374701000
H -0.137973000 1.866377000 -3.454306000
H 0.007584000 3.618901000 -3.176957000
H 1.119535000 2.802546000 -4.298334000
C 4.616697000 1.398297000 -0.936339000
H 5.101338000 1.262886000 -1.902034000
H 4.534662000 0.419852000 -0.463602000
C 5.401660000 2.360148000 -0.063307000
H 5.461389000 3.332585000 -0.560348000
H 4.873968000 2.511772000 0.882567000
C 6.801343000 1.828476000 0.217607000
H 6.722709000 0.848760000 0.698300000
H 7.323772000 1.669068000 -0.730439000
C 7.613135000 2.766343000 1.097973000
H 7.126416000 2.916400000 2.064946000
H 8.610847000 2.364855000 1.285024000
H 7.729515000 3.745681000 0.626912000

56
NO-C4
Ag 1.160970000 -0.345107000 -0.303737000
N -3.386916000 -1.008836000 -0.720909000
S -2.002275000 -1.505273000 -0.214703000
S -4.812433000 -1.499411000 -0.235065000
O -4.815682000 -2.712768000 0.520576000
O -5.736572000 -1.339585000 -1.311159000
O -1.891943000 -1.898179000 1.154489000
O -1.021043000 -0.557344000 -0.713790000
C -1.621856000 -3.029500000 -1.204812000
C -5.298515000 -0.186926000 0.980087000
F -5.240368000 1.019541000 0.427907000
F -4.497308000 -0.208753000 2.040522000
F -6.541376000 -0.413172000 1.382246000
F -1.704603000 -2.767215000 -2.500264000
F -2.459187000 -4.007173000 -0.904807000
F -0.383520000 -3.417369000 -0.921133000
S 4.143825000 -0.949612000 0.974399000
O 5.540818000 -1.053801000 0.714891000
N 3.346080000 0.002974000 -0.043444000
S 4.018403000 1.154926000 -0.938948000
O 3.358828000 -2.126529000 1.168506000
O 2.991365000 2.083868000 -1.291010000
O 5.268911000 1.629547000 -0.448081000
C 4.403435000 0.273104000 -2.527344000
F 5.289112000 -0.689401000 -2.330479000
F 3.299679000 -0.254903000 -3.041632000
F 4.900704000 1.152289000 -3.386017000
C 4.005469000 -0.034792000 2.583814000
F 4.645480000 -0.720578000 3.519118000
F 2.730388000 0.085453000 2.936779000

F	4.536727000	1.174311000	2.490826000
C	-1.705929000	1.787931000	1.346324000
H	-2.684419000	1.456397000	1.645661000
C	-0.068079000	2.653288000	0.181617000
C	0.449403000	2.179706000	1.343203000
H	1.454045000	2.199308000	1.727199000
H	0.403707000	3.147066000	-0.649266000
N	-1.412784000	2.395330000	0.205093000
N	-0.596119000	1.643806000	2.054804000
C	-0.497590000	0.953938000	3.331759000
H	-0.098714000	-0.046666000	3.175989000
H	0.156945000	1.518629000	3.990925000
H	-1.489688000	0.886291000	3.770308000
C	-2.373357000	2.694410000	-0.857897000
H	-2.924763000	1.777482000	-1.067794000
H	-1.788803000	2.944580000	-1.741660000
C	-3.311052000	3.827722000	-0.480173000
H	-3.843191000	3.564507000	0.438527000
H	-2.723733000	4.725672000	-0.267714000
C	-4.316512000	4.110295000	-1.588518000
H	-3.779349000	4.376285000	-2.503791000
H	-4.874706000	3.195621000	-1.809343000
C	-5.285130000	5.223956000	-1.220318000
H	-4.753207000	6.156935000	-1.017251000
H	-5.994104000	5.412346000	-2.028664000
H	-5.858466000	4.964852000	-0.326618000

56
O2-C4

Ag	-0.443455000	0.790582000	-0.886447000
N	-4.212258000	-1.454275000	-0.840859000
S	-3.718982000	-0.009221000	-1.135758000
S	-3.881401000	-2.363925000	0.403524000
O	-4.909214000	-3.342286000	0.547527000
O	-3.423285000	-1.668902000	1.571048000
O	-3.665810000	0.230638000	-2.540476000
O	-2.581662000	0.427374000	-0.343679000
C	-5.082876000	1.075289000	-0.507539000
C	-2.401724000	-3.313879000	-0.183596000
F	-1.386234000	-2.488451000	-0.425878000
F	-2.679475000	-3.983163000	-1.290642000
F	-2.035028000	-4.171816000	0.760311000
F	-5.224473000	0.930382000	0.802505000
F	-6.223087000	0.768832000	-1.103828000
F	-4.780581000	2.339567000	-0.772078000
S	2.742848000	0.136147000	-1.083757000
O	1.756367000	1.191630000	-0.920539000
N	3.652722000	0.147981000	0.171081000
S	4.611002000	-1.000284000	0.687003000
O	2.260106000	-1.136202000	-1.521703000
O	4.264947000	-1.362282000	2.027591000
O	4.894132000	-2.037713000	-0.255184000
C	6.185051000	-0.039284000	0.864548000
F	6.585757000	0.432447000	-0.307634000
F	6.024201000	0.974801000	1.703814000
F	7.121123000	-0.848962000	1.343903000
C	3.776812000	0.785420000	-2.479435000
F	3.010530000	0.936029000	-3.551384000
F	4.750727000	-0.065683000	-2.760520000
F	4.303058000	1.957534000	-2.156163000
C	1.385838000	0.405718000	2.675321000
C	0.684634000	1.554374000	2.815059000
H	2.439491000	0.202463000	2.751142000
H	1.008593000	2.554928000	3.040745000
C	-0.737618000	-0.055030000	2.368984000
H	-1.654290000	-0.587997000	2.165536000
N	-0.639110000	1.243888000	2.619722000
N	0.478497000	-0.585342000	2.394511000
C	0.792820000	-1.984625000	2.149401000
H	1.460022000	-2.341257000	2.930115000
H	-0.130843000	-2.556331000	2.167641000
H	1.273221000	-2.091809000	1.178674000
C	-1.757207000	2.184469000	2.654020000
H	-2.667654000	1.596000000	2.548949000
H	-1.765194000	2.653642000	3.638237000
C	-1.656627000	3.226913000	1.554633000
H	-0.728904000	3.794239000	1.668304000

H -1.606365000 2.718298000 0.586454000
C -2.847392000 4.175086000 1.572231000
H -3.771644000 3.593635000 1.513194000
H -2.872854000 4.703682000 2.529777000
C -2.800922000 5.179864000 0.431425000
H -3.664506000 5.846866000 0.458083000
H -1.899895000 5.796019000 0.487090000
H -2.799264000 4.673424000 -0.536969000

56
NO2-C4
Ag -0.651025000 -0.587810000 0.655652000
N 3.052379000 -1.328580000 -0.110957000
S 2.775699000 -1.044483000 1.397327000
S 2.075057000 -1.921419000 -1.190171000
O 0.749704000 -2.220412000 -0.713805000
O 2.737461000 -2.900057000 -1.987431000
O 1.395140000 -0.805851000 1.756263000
O 3.743667000 -0.124339000 1.901364000
C 3.163842000 -2.655547000 2.226338000
C 1.846958000 -0.465316000 -2.312524000
F 2.995863000 -0.118013000 -2.871101000
F 1.367672000 0.572632000 -1.633718000
F 0.983164000 -0.788568000 -3.264191000
F 4.423006000 -2.996283000 2.000662000
F 2.367269000 -3.609055000 1.761567000
F 2.975192000 -2.532877000 3.532811000
S -3.891833000 -0.213373000 1.187648000
O -3.293791000 -0.886723000 2.296770000
N -2.777091000 -0.090929000 0.039522000
S -3.098734000 0.134872000 -1.517241000
O -5.183995000 -0.601230000 0.728406000
O -4.371004000 0.724634000 -1.774579000
O -1.926024000 0.683227000 -2.122479000
C -3.204943000 -1.589876000 -2.199241000
F -2.058385000 -2.229088000 -2.027275000
F -4.172824000 -2.272222000 -1.608495000
F -3.462997000 -1.506803000 -3.497582000
C -4.083279000 1.538930000 1.771394000
F -4.982973000 1.565583000 2.743834000
F -4.482313000 2.332997000 0.790610000
F -2.927939000 1.994786000 2.244965000
C 1.199022000 3.780086000 -0.323103000
H 1.321961000 4.415198000 -1.183832000
C 1.631895000 2.511621000 1.409888000
C 0.288054000 2.580044000 1.268443000
H -0.503297000 2.157492000 1.860683000
H 2.241748000 2.004043000 2.135655000
N 2.180837000 3.270208000 0.409116000
N 0.040172000 3.378789000 0.179769000
C -1.276871000 3.727047000 -0.329029000
H -1.756320000 2.836455000 -0.729417000
H -1.873191000 4.145124000 0.478837000
H -1.162670000 4.463874000 -1.120300000
C 3.612495000 3.423207000 0.150684000
H 4.114937000 3.342366000 1.114415000
H 3.764878000 4.431491000 -0.230578000
C 4.117876000 2.363747000 -0.816935000
H 3.804398000 1.383552000 -0.452279000
H 3.641697000 2.515100000 -1.789846000
C 5.634651000 2.380144000 -0.970956000
H 6.095113000 2.184140000 0.002037000
H 5.913945000 1.545156000 -1.617402000
C 6.189504000 3.674462000 -1.551316000
H 6.032572000 4.522464000 -0.881052000
H 7.264057000 3.591514000 -1.725174000
H 5.714188000 3.910018000 -2.507283000

56
O4-C4
Ag -0.103569000 0.322108000 -0.773465000
N 3.232467000 -1.481536000 -0.931549000
S 3.352878000 0.002501000 -1.381768000
S 2.299134000 -2.140006000 0.144589000
O 1.223556000 -1.307167000 0.623072000
O 3.052193000 -2.864728000 1.115662000
O 2.183460000 0.823803000 -1.164825000

O	3.980185000	0.080550000	-2.659561000
C	4.587915000	0.704335000	-0.193691000
C	1.469110000	-3.429712000	-0.892678000
F	2.364106000	-4.225080000	-1.457103000
F	0.740285000	-2.856252000	-1.842650000
F	0.675740000	-4.155298000	-0.117128000
F	5.756126000	0.098303000	-0.325863000
F	4.160353000	0.544949000	1.055618000
F	4.745813000	2.001512000	-0.430214000
S	-3.075201000	-0.591102000	1.082639000
O	-3.873026000	-0.481318000	2.259567000
N	-3.975927000	-0.413805000	-0.187638000
S	-3.576990000	0.082061000	-1.616427000
O	-1.822708000	0.122854000	1.056930000
O	-2.167088000	0.050995000	-1.927328000
O	-4.469741000	-0.459173000	-2.586718000
C	-3.975884000	1.890738000	-1.559378000
F	-5.252748000	2.078957000	-1.268438000
F	-3.231426000	2.500880000	-0.641781000
F	-3.716372000	2.430912000	-2.743131000
C	-2.579108000	-2.373900000	0.989854000
F	-1.757222000	-2.652472000	1.991488000
F	-1.957372000	-2.613004000	-0.158011000
F	-3.644158000	-3.156425000	1.072216000
C	1.951582000	3.109701000	1.151025000
H	2.951592000	3.054696000	1.545380000
C	-0.195146000	2.784161000	0.861042000
C	0.242475000	3.602759000	-0.127235000
H	-0.283216000	4.071763000	-0.940479000
H	-1.178607000	2.417708000	1.094423000
N	0.893985000	2.486713000	1.646605000
N	1.584351000	3.794781000	0.076494000
C	2.470774000	4.560429000	-0.785393000
H	3.416238000	4.713287000	-0.272070000
H	2.636615000	4.012861000	-1.711548000
H	2.012209000	5.523016000	-1.000265000
C	0.906852000	1.540113000	2.764058000
H	1.926217000	1.520368000	3.147421000
H	0.681817000	0.559150000	2.347377000
C	-0.077050000	1.918706000	3.857025000
H	0.186030000	2.907283000	4.241288000
H	-1.084000000	1.988755000	3.436323000
C	-0.079372000	0.900197000	4.994917000
H	0.934341000	0.803395000	5.396104000
H	-0.694682000	1.300149000	5.804040000
C	-0.610118000	-0.471761000	4.595602000
H	0.018576000	-0.954763000	3.844959000
H	-0.651528000	-1.135321000	5.461585000
H	-1.620045000	-0.393716000	4.186094000
